# The safety of double- and triple-drug community mass drug administration for lymphatic filariasis: A multicenter, open-label, cluster-randomized study

**DOI:** 10.1371/journal.pmed.1002839

**Published:** 2019-06-24

**Authors:** Gary J. Weil, Joshua Bogus, Michael Christian, Christine Dubray, Yenny Djuardi, Peter U. Fischer, Charles W. Goss, Myra Hardy, Purushothaman Jambulingam, Christopher L. King, Vijesh Sridhar Kuttiat, Kaliannagounder Krishnamoorthy, Moses Laman, Jean Frantz Lemoine, Katiuscia K. O’Brian, Leanne J. Robinson, Josaia Samuela, Kenneth B. Schechtman, Anita Sircar, Adinarayanan Srividya, Andrew C. Steer, Taniawati Supali, Swaminathan Subramanian, the DOLF IDA Safety Study Group

**Affiliations:** 1 Washington University, St. Louis, Missouri, United States of America; 2 Universitas Indonesia, Jakarta, Indonesia; 3 Centers of Disease Control and Prevention, Atlanta, Georgia, United States of America; 4 Murdoch Children’s Research Institute, Melbourne, Australia; 5 ICMR-Vector Control Research Centre, Puducherry, India; 6 Case Western Reserve University, Cleveland, Ohio, United States of America; 7 Papua New Guinea Institute of Medical Research, Madang, Papua New Guinea; 8 Ministère de la Santé Publique et de la Population (MSPP), Port-au-Prince, Haïti; 9 Burnet Institute, Melbourne, Australia; 10 Fiji Ministry of Health and Medical Services, Suva, Fiji; Liverpool School of Tropical Medicine, UNITED KINGDOM

## Abstract

**Background:**

The Global Programme to Eliminate Lymphatic Filariasis (GPELF) provides antifilarial medications to hundreds of millions of people annually to treat filarial infections and prevent elephantiasis. Recent trials have shown that a single-dose, triple-drug treatment (ivermectin with diethylcarbamazine and albendazole [IDA]) is superior to a two-drug combination (diethylcarbamazine plus albendazole [DA]) that is widely used in LF elimination programs. This study was performed to assess the safety of IDA and DA in a variety of endemic settings.

**Methods and findings:**

Large community studies were conducted in five countries between October 2016 and November 2017. Two studies were performed in areas with no prior mass drug administration (MDA) for filariasis (Papua New Guinea and Indonesia), and three studies were performed in areas with persistent LF despite extensive prior MDA (India, Haiti, and Fiji). Participants were treated with a single oral dose of IDA (ivermectin, 200 μg/kg; diethylcarbamazine, 6 mg/kg; plus albendazole, a fixed dose of 400 mg) or with DA alone. Treatment assignment in each study site was randomized by locality of residence. Treatment was offered to residents who were ≥5 years of age and not pregnant. Adverse events (AEs) were assessed by medical teams with active follow-up for 2 days and passive follow-up for an additional 5 days. A total of 26,836 persons were enrolled (13,535 females and 13,300 males). A total of 12,280 participants were treated with DA, and 14,556 were treated with IDA. On day 1 or 2 after treatment, 97.4% of participants were assessed for AEs. The frequency of all AEs was similar after IDA and DA treatment (12% versus 12.1%, adjusted odds ratio for IDA versus DA 1.15, 95% CI 0.87–1.52, *P* = 0.316); 10.9% of participants experienced mild (grade 1) AEs, 1% experienced moderate (grade 2) AEs, and 0.1% experienced severe (grade 3) AEs. Rates of serious AEs after DA and IDA treatment were 0.04% (95% CI 0.01%–0.1%) and 0.01% (95% CI 0.00%–0.04%), respectively. Severity of AEs was not significantly different after IDA or DA. Five of six serious AEs reported occurred after DA treatment. The most common AEs reported were headache, dizziness, abdominal pain, fever, nausea, and fatigue. AE frequencies varied by country and were higher in adults and in females. AEs were more common in study participants with microfilaremia (33.4% versus 11.1%, *P* < 0.001) and more common in microfilaremic participants after IDA than after DA (39.4% versus 25.6%, *P* < 0.001). However, there was no excess of severe or serious AEs after IDA in this subgroup. The main limitation of the study was that it was open-label. Also, aggregation of AE data from multiple study sites tends to obscure variability among study sites.

**Conclusions:**

In this study, we observed that IDA was well tolerated in LF-endemic populations. Posttreatment AE rates and severity did not differ significantly after IDA or DA treatment. Thus, results of this study suggest that IDA should be as safe as DA for use as a MDA regimen for LF elimination in areas that currently receive DA.

**Trial registration:**

Clinicaltrials.gov registration number: NCT02899936

## Abstract 2

In an open-label, cluster-randomized study done in Papua New Guinea, Indonesia, India, Haiti, and Fiji, Gary J. Weil and team assess the safety of double- and triple-drug community mass drug administration for lymphatic filariasis.

## Author summary

**Why was this study done?:**

- Lymphatic filariasis (LF, also known as “elephantiasis”) is a major global health problem caused by nematode worms that are transmitted by mosquitoes. The World Health Organization’s Global Programme to Eliminate Lymphatic Filariasis aims to eliminate LF from all areas with the disease with repeated annual rounds of mass treatment with medicines that kill the parasites and reduce transmission of new infections.
- Small clinical trials have shown that treatment with a single dose of a new triple-drug combination (“IDA”, comprising ivermectin with diethylcarbamazine and albendazole [IDA]) is superior to treatment with a single dose of the standard two-drug combination (diethylcarbamazine plus albendazole [DA] alone). IDA might accelerate LF elimination in many countries.
- However, a more effective treatment might be associated with more frequent or severe adverse events (AEs). This study was performed to assess and compare the safety of mass treatment with IDA and DA in a variety of settings.

**What did the researchers do and find?:**

- We performed large community treatment studies to compare the safety of the three-drug and two-drug treatments in five countries (India, Haiti, Indonesia, Papua New Guinea, and Fiji).
- More than 26,000 people were tested for LF infection, treated, and assessed for AEs after treatment. About 12% of study participants developed AEs, and this rate was the same after treatment with either IDA or DA.
- AEs were more common in persons who had filarial worms in their blood before treatment and more common after IDA than after DA in this subgroup. However, there was no excess of severe or serious AEs after IDA.

**What do these findings mean?:**

- Results from this study show that the triple-drug combination IDA was well tolerated in a variety of filariasis-endemic settings. IDA should be safe for use as a mass treatment regimen for LF elimination in areas that currently receive the double-drug combination DA.
- IDA has the potential to accelerate LF elimination in many countries if public health programs can achieve high rates of mass drug administration adherence.

## Introduction

Lymphatic filariasis (LF) is a disabling and deforming neglected tropical disease caused by filarial nematode parasites (*Wuchereria bancrofti*, *Brugia malayi*, and *B*. *timori*) that are transmitted by mosquitoes. Adult filarial worms reside in lymphatic vessels; the adult worms (and host inflammatory responses to them) lead to lymphedema, elephantiasis, and hydroceles. The Global Programme to Eliminate Lymphatic Filariasis (GPELF, coordinated by the World Health Organization [WHO]) was launched in the year 2000 with the goal of eliminating LF as a public health problem by 2020 [1,2]. At that time, some 80 countries were considered to be endemic for LF, and the target population at risk for infection was more than 1.3 billion. GPELF’s elimination strategy is largely based on repeated rounds of mass administration of antifilarial drugs (MDA) to populations at risk for infection. These drugs temporarily clear microfilariae (Mf) from the blood (preventing uptake by mosquitoes), and they have partial macrofilaricidal activity (the ability to kill adult filarial worms). Drug donations from pharmaceutical companies allowed GPELF to quickly expand, and progress has been very significant [3–5]. Between 2000 and 2016, some 6.7 billion treatments were delivered to more than 850 million people. In addition, the number of countries that required additional MDA was reduced to 52, and the population at risk for infection was reduced by approximately 40% [5]. GPELF is arguably the largest control or elimination program for an infectious disease to date based on MDA.

Despite this progress, GPELF faces many challenges, and the 2020 target date for global LF elimination will not be met. Some endemic areas have not yet received a single round of MDA, and many areas around the world have not met elimination targets despite having received five or more rounds of MDA. Elimination programs using the currently recommended MDA regimens (diethylcarbamazine [DEC] plus albendazole [DA] in most endemic countries, with ivermectin plus albendazole [IA] in sub-Saharan African countries that are coendemic for onchocerciasis or loiasis) require at least five annual, effective rounds of MDA to reach elimination targets [6]. Multiple MDA rounds are required, because these treatments have limited ability to kill or permanently sterilize adult filarial worms. Repeated MDA rounds also increase cumulative compliance, the percentage of the population at risk that has swallowed at least one dose of the treatment. Recent clinical trials have shown that a single dose of a triple-drug combination comprising of ivermectin with DEC and albendazole (IDA) is superior to IA or DA for clearing larval filarial parasites (Mf) from the blood [7–9]. Since Mf are essential for transmission of LF by vector mosquitoes, computer simulation studies suggest that substitution of IDA for the two-drug MDA regimens could significantly accelerate LF elimination if compliance is high and systematic noncompliance is low [10].

All three drugs in the IDA treatment regimen have wide safety margins in uninfected individuals, and billions of doses of these drugs have been administered by LF elimination programs. However, a recent review of the LF treatment literature reported that mild to moderate adverse events (AEs) were common following treatment of LF with single- or double-drug regimens [11]. Although reported AE rates after LF treatment varied widely, they tended to be higher in clinical trials that required microfilaremia for inclusion than in community treatment studies that typically enroll many persons who are not infected. Also, AE rates reported from community treatment studies tended to be higher in studies with active follow-up of participants than in studies that used passive assessment of AEs based on participants presenting for management of symptoms [11].

Systemic AEs that occur after treatment with antifilarial drugs (e.g., fever, headache, and myalgia) are believed to be triggered by the death of Mf, and the risk of systemic AEs following treatment is related to blood Mf counts [11,12]. A more effective treatment such as IDA might be expected to be associated with a higher rate of AEs. Indeed, mild to moderate AEs were more common after IDA treatment than after IA or DA in clinical trials [7,9]. More safety data were needed before IDA could be recommended for widespread use. Therefore, this study was performed to assess the safety of IDA and to compare the safety for individuals of MDA with IDA versus DA on a large scale in different LF-endemic settings; this was accomplished with a cluster-randomized trial.

## Methods

### Overview

The Clinicaltrials.gov registration number for this study is NCT02899936. This study is reported as per the Consolidated Standards of Reporting Trials (CONSORT) guideline. The primary objective of the study was to compare the frequency, type, and severity of AEs following community MDA with IDA or DA. We were especially interested in comparing rates of serious AEs (SAEs) following IDA and DA. This is because WHO procedure for policy change places a high priority on having data to show that treatment-related SAE rates are no higher than 0.1% for new treatments. The study also aimed to assess the effect of filarial infection on the frequency and severity of AEs, to compare the efficacy of these drug combinations for clearing Mf and circulating filarial antigenemia (CFA), and to compare the community acceptance of MDA by treatment group. This paper presents an overview of consolidated safety results from all five study sites. Detailed results from individual countries and results of acceptability and efficacy studies will be reported separately.

The study was performed in five countries with the same basic protocol, although minor modifications were permitted to account for local institutional review board (IRB) requirements. Three studies (in India, Fiji, and Haiti) were conducted in areas that had persistent LF with Mf rates above 1% despite seven or more annual rounds of MDA with DA. In contrast, two studies (in Indonesia and in Papua New Guinea) were conducted in LF-endemic areas that had no prior history of MDA. The predominant filarial species in the Indonesia study sites is *B*. *timori*, but *W*. *bancrofti* is also present. All other sites were endemic for *W*. *bancrofti* only. A representative protocol from the study is provided as supplemental file S1 Appendix.

### Site preparation

Preliminary meetings were held with ministry of health personnel at different levels. Study site selection was based on a number of factors including presence or persistence of LF, the presence of a regulatory environment for adequate consideration of ethical issues that was amenable to importation of study medications, and availability of personnel required to conduct the study. Prior to initiating each study, research teams visited the study area to meet with local health officials, community leaders, and staff at local health centers and hospitals. These meetings were important for planning and conducting social mobilization activities to increase community participation in the study. Study staff were trained in good clinical practice (GCP) and on project-specific standard operating procedures with an emphasis on informed consent, parasitology testing, and data capture and transmission [13]. Medical personnel were trained on the project goals and protocol with an emphasis on clinical evaluation, management, and reporting of AEs.

### Ethical review and oversight

The study protocols were reviewed and approved by independent Federal-Wide Assurance (FWA) registered ethical review boards in each country and at institutions of research partners who participated in the studies. These included the Human Research Protection Office, Washington University School of Medicine, St. Louis, MO, United States; University Hospitals of Cleveland IRB, Cleveland, OH, USA; Comité National de Bioéthique, Ministère de la Santé Publique et de la Population, Port-au-Prince, Haiti; Fiji National Health Research and Ethics Review Committee, Suva, Fiji; Royal Children’s Hospital Human Research Ethics Committee, Melbourne, Australia; Indian Council of Medical Research Institutional Human Ethics Committee, VCRC, Puducherry, India; Government of Papua New Guinea Medical Research Advisory Committee, Waigani, Papua New Guinea IRB, Papua New Guinea Institute of Medical Research, Goroka, Papua New Guinea; and The Ethics Committee of the Faculty of medicine, University of Indonesia, Jakarta, Indonesia.

Safety data were reviewed periodically by a single data safety monitoring board (DSMB) based in the USA. Independent medical monitors in each country were responsible for assessing SAE reports and for consulting with attending physicians to determine whether SAEs were related to the study drugs. Medical monitors in most study sites were government physicians based at district hospitals that serve study villages. They were provided GCP training and training on grading of AEs and on the definition of “serious adverse events” [14]. The medical monitor for the India study was a clinical pharmacologist and a medical school faculty member with extensive clinical trial experience.

### Treatment assignment

This was an open-label, cluster-randomized trial. Project statisticians used a random number generator to assign treatment regimens (DA or IDA) by cluster. Clusters were villages in India, Papua New Guinea, and Fiji; neighborhoods in Haiti; and subvillages (dusuns) in Indonesia. Villages in India and in Papua New Guinea and dusuns in Indonesia were block randomized by population size and infection prevalence. Infection prevalence was not known for clusters in other study sites prior to randomization. Cluster sizes varied within study sites and between different country study sites.

### Source of medications and treatment

Ivermectin was donated by Merck Sharp Dohme (MSD), also known as Merck & Co., (Kenilworth, NJ, USA), for all study sites except Fiji. Ivermectin was purchased from MSD (Australia) for the Fiji study. Albendazole (produced and donated by GlaxoSmithKline) and DEC (produced and donated by Eisai Co.) were obtained from ministry of health stocks in each country. Ingestion of study medications was directly observed by project staff at all sites. All study sites used a fixed dose of 400 mg for albendazole for all participants. Four studies provided weight-based treatment for ivermectin (200 μg/kg) and DEC (6 mg/kg); the number of tablets provided was based on a weight-based dosing table. The India study used weight-based dosing for ivermectin, but it employed age-based dosing for DEC according to national guidelines, with a maximum dose of three 100-mg tablets. The Fiji study was designed to compare the impact of DA and IDA on both LF and scabies. Therefore, that study included two IDA treatment arms, one of which received a second dose of ivermectin 8 days after the first treatment. This explains the excess number of people treated with IDA in Fiji. The AE assessment data reported here for Fiji ended at 7 days as in the other study sites.

### Enrollment

Enrollment teams included a physician and/or a nurse, a technician, and a local assistant. Teams visited each house in the cluster (India and Haiti) or used temporary stations to enroll residents from closely spaced household clusters (Indonesia, Fiji, Papua New Guinea). Exclusion criteria were age < 5 years, weight less than 15 kg, pregnancy (or last menstrual period > 4 weeks ago or unknown), breastfeeding within 7 days of delivery, acute or chronic illness severe enough to interfere with activities of daily living, or any history of previous allergy to the study drugs. Enrollment teams used a preprinted script to explain the purpose of the study and study procedures to household or community members. They then obtained written informed consent from individuals. Participation of children required written informed consent from at least one parent and assent from the child.

### Parasitology tests

Samples for parasitology testing were collected just prior to treatment. CFA in finger prick blood was detected with Alere Filariasis Test Strips (FTS) (Alere Scarborough, Scarborough, ME, USA) according to the manufacturer’s protocol. Test results were scored essentially as previously described [15], based on the intensity of the test (“T”) line. Test scores were recorded as follows: 0, no test line visible (a negative test); 1, the test line is present but weaker than the control line; 2, the test line is equal in intensity to the control line; 3, the test line is stronger than the control line. Tests with no control line were considered to have invalid results. CFA is a marker for infection with *W*. *bancrofti* adult worms. A subset of persons with positive CFA tests have microfilaremia. Participants with positive antigen tests were tested for microfilaremia with 60-μl-thick smears prepared with blood collected by finger prick between the hours of 9 PM and 1 AM, as previously described [15, 16], or during the day in Fiji, which has subperiodic bancroftian filariasis. In the Indonesia study site, where *B*. *timori* is predominant and not detected by FTS, night blood was collected from all participants for Mf testing by thick blood smear. Stained smears were examined by experienced microscopists; Mf species were identified by morphology.

### Assessment and management of AEs

Field teams attempted to visit all treated participants for AE assessment on days 1 and 2 after treatment. Passive monitoring was performed to detect late-onset AEs extended to 7 days after treatment, and participants with an AE of severity of 2 or higher by the second day after treatment were followed until their symptoms were mild or resolved. For passive monitoring, AE assessment teams visited persons with late-onset AEs identified by study staff based in the village and/or by staff at local primary health centers that serve residents of study villages. A modified version of the document “Common Terminology Criteria for Adverse Events” (CTCAE) Version 4.03 was used to categorize and score AEs [17] (S1 Table). Briefly, grade 1 AEs were mild and did not prevent the participant from working or going to school. Grade 2 AEs were moderate and interfered with work or school attendance. Participants with AEs with severity of grade 3 or higher that interfered with activities of daily living were referred for evaluation by a physician who was not part of the research team, who documented the nature of the problem and determined whether it required hospitalization or otherwise met criteria for an SAE [14]. Physicians also recorded whether they thought that SAEs were related to study medications. Study teams provided participants with acetaminophen and/or ibuprofen as needed for fever or pain and oral rehydration in persons with dehydration.

### Data acquisition, transfer, and management

An electronic data capture (EDC) system developed by CliniOps (Fremont, CA, USA) was used to standardize data collection across the study sites and to compile the data into one complete dataset. Deidentified data were entered directly into a tablet via a mobile data management application called CliniTrial. The data were entered by a designated member of each survey team on the day of enrollment or AE assessment. The EDC system is 21 CFR Part 11 compliant. Electronic case report forms (CRFs) were developed to comply with International Council for Harmonization on Good Clinical Practice (ICH GCP) and CDASH/CDISC standards. Extensive user acceptability testing was performed for CRFs prior to their deployment. Validation checks and automated alert checks were programmed into the EDC system to maintain a high level of data quality at point of entry. Data were synced regularly through a secured server. AEs were coded using MedDRA dictionaries (version 20.0) [18]. Paper CRFs were used for backup in case of EDC or equipment malfunction and for documentation of SAEs. All written forms (i.e., consent and backup data collection forms) were stored at the endemic country collaborator’s institution as per in-country IRB requirements for storage of source documents.

### Statistical methods and sample size

The statistical analysis plan for this study is provided as supplemental file S2 Appendix. WHO guidelines for changes in treatment policy recommend cohort event monitoring studies with at least 10,000 participants to provide high confidence that the rate of SAEs for a new treatment is less than 0.1% [19], and our study was powered according to this recommendation. The high sample size provides an additional margin of safety, and it allows for the fact that it is sometimes difficult to interpret the meaning of a single SAE. In addition to documenting the rate of SAEs after IDA, an important secondary objective of the data analysis was to determine whether the triple-drug regimen was associated with an increased risk of AEs compared to the double-drug regimen. The outcome of interest was an AE of any severity within the 7-day monitoring period (AE present). This outcome was considered at the level of the individual participant and not at the level of study site or cluster. We used a generalized linear mixed model (GLMM) approach to analyze these dichotomous outcome data assuming a binomial probability distribution and a logit link function (PROC GLIMMIX [SAS Institute, Cary, NC, USA] [20]). Locality within study sites was treated as a random effect to account for correlation among subjects within a locality. The drug treatment effect was adjusted by country, Mf and/or filarial antigen test positivity, age group (children [< 18 years] and adults [≥ 18 years]), and sex. We conducted both univariable and multivariable analyses to obtain estimates of treatment effects, both adjusted and unadjusted for covariates. All other dichotomous outcome analyses such as crude AE rates were conducted using simple chi-squared tests. Mean Mf counts (number of Mf per 60 μl blood) were compared between subjects who experience an AE versus those that did not experience an AE using a linear mixed model that included a random locality nested within study site effect. The Mf count outcome was log transformed, and group estimates are presented as geometric means with 95% CIs (antilog [model-adjusted mean, 95% CI]). *P* values < 0.05 were considered significant.

## Results

### Enrollment and filarial infection rates by study site

**Fig 1:**
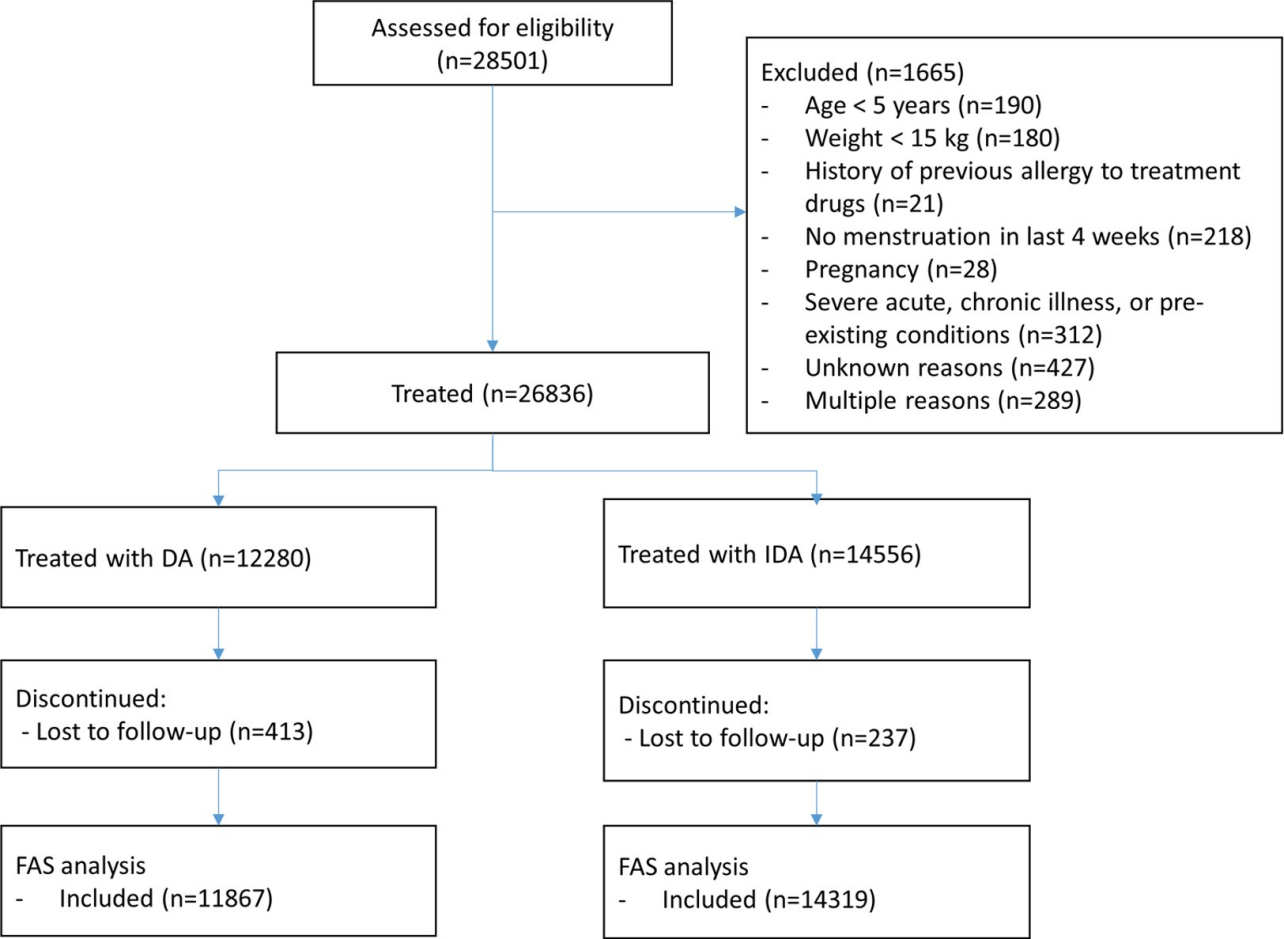
CONSORT diagram. Treatment allocation was by cluster randomization at each study site. DA, diethylcarbamazine plus albendazole; FAS, full analysis set; IDA, ivermectin plus diethylcarbamazine and albendazole.

A total of 26,836 participants were enrolled and treated in five countries. Enrollment started in India in October 2016 and ended in Fiji in November 2017. A CONSORT diagram is provided in Fig 1, and a CONSORT checklist is provided in supplemental file S1 CONSORT Checklist. Baseline filarial infection rates are shown by country in Table 1.

**Table 1:** Filarial infection prevalence in the study sites.

Site	District	Mf Prevalence^a^	CFA^a^ Prevalence
Fiji	Gau	33/1,957 (1.7%)	124/1,957 (6.3%)
	Rotuma	106/1,454 (7.3%)	378/1,469 (25.7%)
Haiti	Northern Dept	114/5,987 (1.9%)	583/5,993 (9.7%)
India	Yadgir	591/8,825 (6.7%)	2,275/8,887 (25.6%)
Indonesia	Flores	20/1,254 (1.6%)	54/1,249 (4.3%)
	Sumba	94/2,667 (3.5%)	212/2,655 (8.0%)
PNG	Bogia	199/4,518 (4.4%)	1,013/4,550 (22.3%)

^a^There were missing values for Mf and CFA, and therefore the denominators are not equal to the total number of participants treated.

Abbreviations: CFA, circulating filarial antigenemia; Dept, Département; Mf, microfilaremia; PNG, Papua New Guinea.

**Fig 2:**
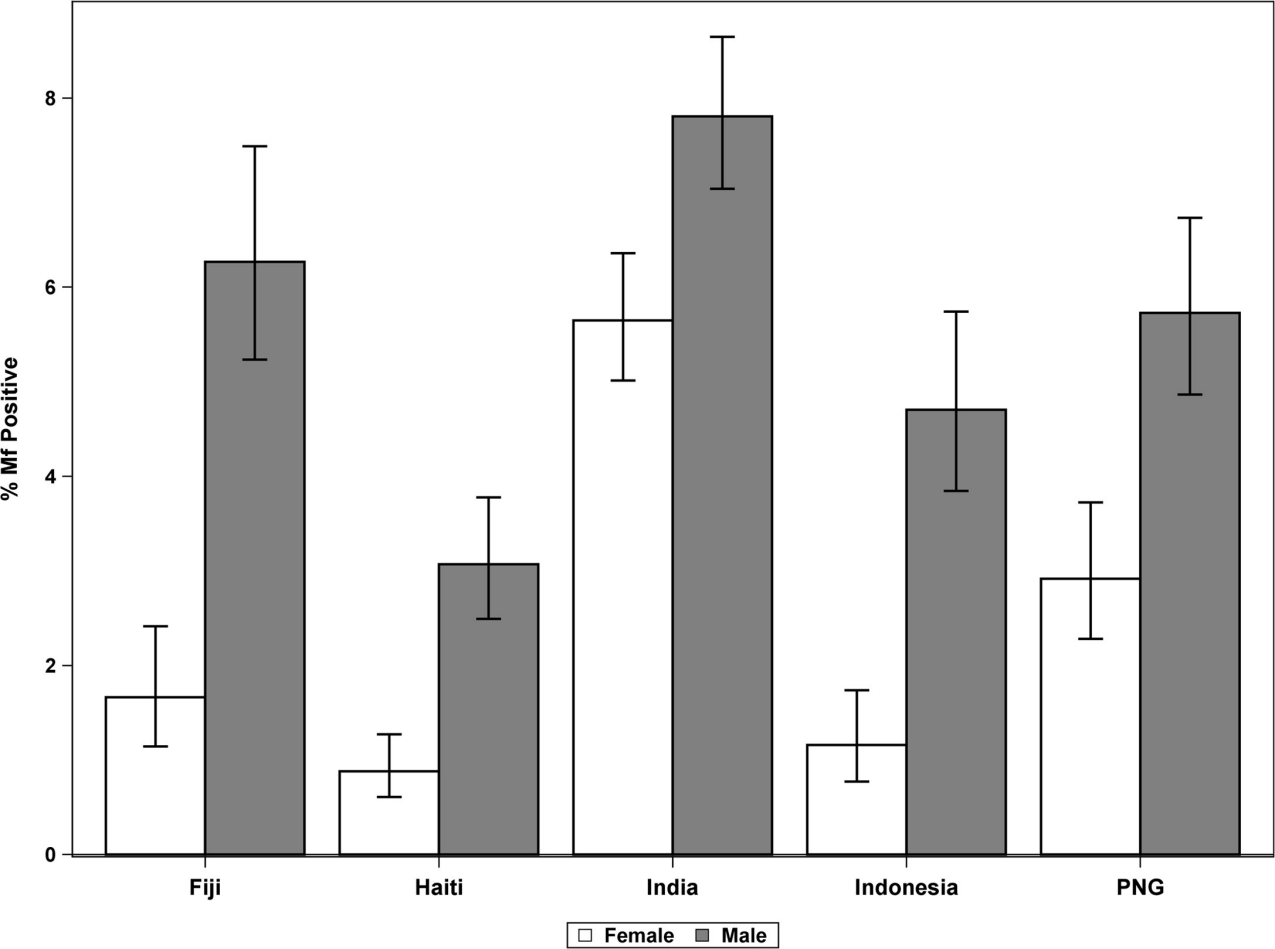
Mf prevalence (%) by sex and country. Error bars indicate 95% CIs. CI, confidence interval; Mf, microfilaremia; PNG, Papua New Guinea.

The two treatment groups were very similar with respect to basic demographics and infection status (Table 2). Males had significantly higher Mf prevalence than females had in all study sites (overall 5.8% and 2.9%, respectively, *P* < 0.001; Fig 2).

**Table 2:** Demographic information and other characteristics of enrolled subjects by treatment group.

Characteristic	DA	IDA
**Enrollment**^a^	12,280	14,556
Haiti	2,994 (24.4)	3,004 (20.6)
India	4,160 (33.9)	4,758 (32.7)
Indonesia	1,786 (14.5)	2,140 (14.7)
PNG	2,181 (17.8)	2,382 (16.4)
Fiji	1,159 (9.4)	2,272 (15.6)
**Sex**^a^		
Female	6,206 (50.5)	7,330 (50.4)
**Age group**^a^		
Adults	7,214 (58.7)	8,646 (59.4)
**Age (years)** ^b^		
Mean	25.8 ± 16.7	26.3 ± 16.8
**Infection status**^a^		
Microfilaremia positive^c^	533 (4.4)	624 (4.3)
CFA positive^d^	2,142 (17.5)	2,497 (17.2)

^a^Reported as *n* (%).

^b^Reported as mean ± SD.

^c^*n* = 12,197 DA; 14,465 IDA.

^d^Denominators exclude persons with missing CFA results. *n* = 12,243 DA; 14,517 IDA.

Abbreviations: CFA, circulating filarial antigenemia; DA, double-drug therapy (diethylcarbamazine, albendazole); IDA, triple-drug therapy (ivermectin, diethylcarbamazine, albendazole); PNG, Papua New Guinea.

### AE assessment, AE rates, and risk factors for AEs

**Fig 3:**
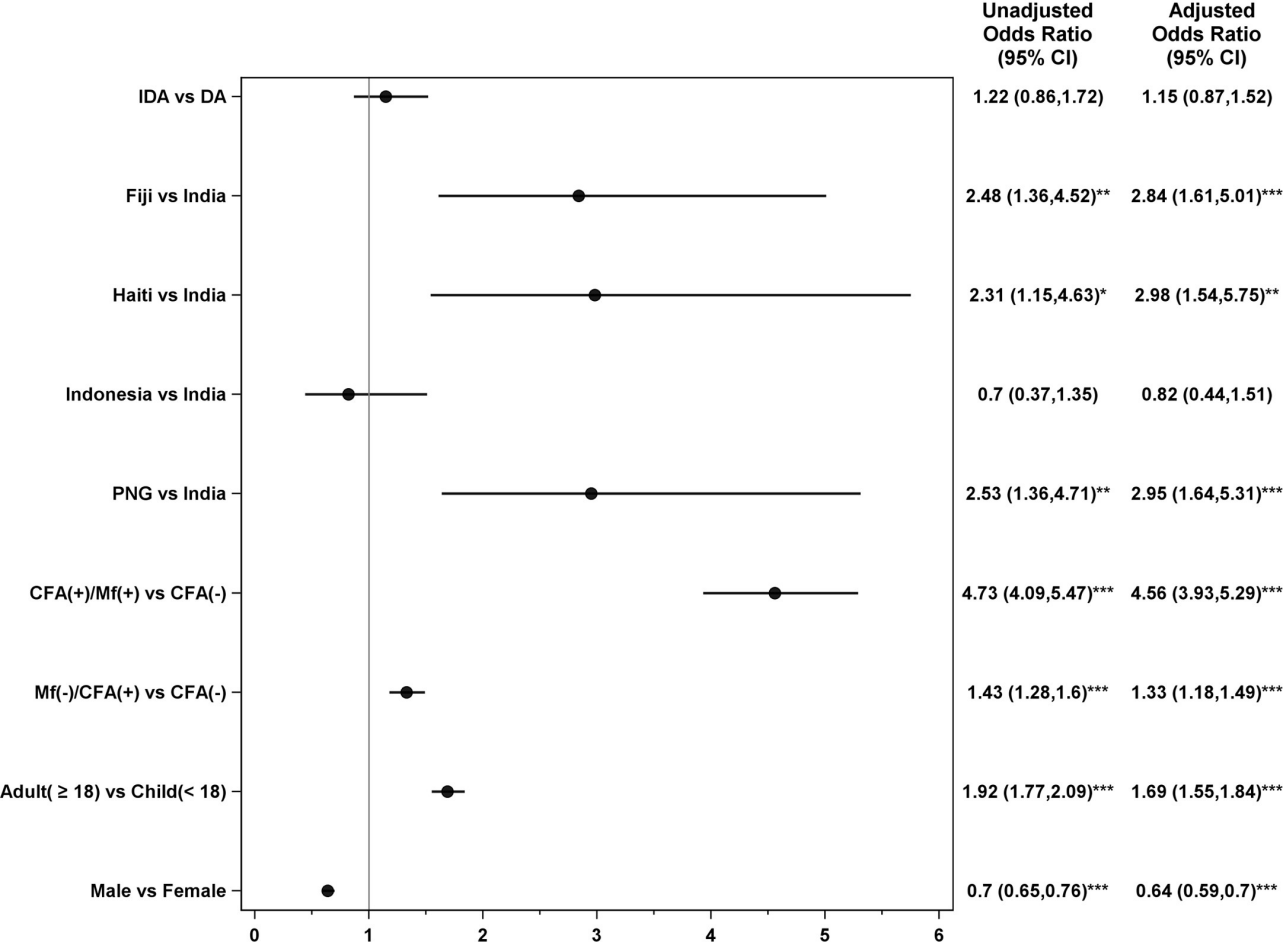
Forest plot showing adjusted odds ratios for factors associated with AEs following treatment for lymphatic filariasis. Odds ratios were assessed relative to the listed reference groups. *P* values for comparisons to reference group: *<0.05, **<0.01, ***<0.001. Note that both unadjusted and adjusted models contain a random effect to account for correlation among subjects within a locality. Only the adjusted odds ratios and 95% CIs are plotted. All univariable models had a total *N* of 26,186 (3,147 with AEs) except for the infection group model, which had an *N* of 25,978 (3,122 with AEs) because of missing values for CFA or Mf. The multivariable model excluded all subjects with missing values for a total *N* of 25,978. Indonesian participants with Mf were considered to be CFA(+) for this analysis, although sometimes this was not the case. That is because *Brugia* infections are not detected by the CFA test. (-), negative results; (+), positive results; AE, adverse event; CFA, circulating filarial antigenemia; CI; confidence interval; DA, double-drug therapy (albendazole, diethylcarbamazine); IDA, triple-drug therapy (albendazole, diethylcarbamazine, ivermectin); MF, microfilaremia; PNG, Papua New Guinea.

The study achieved high participant follow-up rates for AE assessment (S2 Table). Approximately 97.4% of participants were assessed on day 1 and/or day 2 after treatment. Overall, 12.0% of all participants experienced AEs (12% after IDA and 12.1% after DA), and there were no statistically significant differences in overall AE rates or severity of AEs by treatment regimen (Table 3 and Fig 3). The intracluster correlation coefficient for AEs was low (0.07). AE rates in amicrofilaremic participants were not significantly different after treatment with DA or IDA (*P* = 0.09, Table 3). AEs were more common in persons with microfilaremia than in uninfected persons or in persons with isolated filarial antigenemia without microfilaremia (Table 3 and Fig 3). For persons with microfilaremia, pretreatment Mf counts (by 60 μl blood smear) were significantly higher in persons who experienced AEs after treatment (geometric means: 15.7 [95% CI 12.4–19.8] versus 6.7 [5.5–8.3], *P* < 0.001). Although AE rates in persons with Mf were higher after IDA treatment (39.4%) than after DA (25.6%) (*P* < 0.001 by chi-squared test), only mild and moderate AEs were increased (grades 1 or 2, Table 3). AE rates in persons with FTS test scores of 1, 2, or 3 were 16.8%, 16.5%, and 20.7%, respectively. Mf prevalence in persons with FTS scores of 1, 2, or 3 were 7.3%, 13.7%, and 40%, respectively.

**Table 3:** AEs by treatment regimen and infection status.

Drug Regimen	Mf Test Results^a^	Total Number of People Treated and Assessed for AEs and for Mf	Any AE *n* (%)	Grade 1 *n* (%)	Grade 2 *n* (%)	Grade 3 *n* (%)	Grade 4 *n* (%)	SAE *n* (%)
**DA**	**Mf(−)**	11,262	1,287 (11.4)	1,176 (10.4)	99 (0.9)	9 (0.1)	0 (0.0)	3 (0.0)
	**Mf(+)**	524	134 (25.6)	124 (23.7)	7 (1.3)	1 (0.2)	0 (0.0)	2 (0.4)
	**Total**	11,786	1,421 (12.1)	1,300 (11.0)	106 (0.9)	10 (0.1)	0 (0.0)	5 (0.0)
**IDA**	**Mf(−)**	13,617	1,464 (10.8)	1,340 (9.8)	113 (0.8)	10 (0.1)	0 (0.0)	1 (0.0)
	**Mf(+)**	612	241 (39.4)	205 (33.5)	35 (5.7)	1 (0.2)	0 (0.0)	0 (0.0)
	**Total**	14,229	1,705 (12.0)	1,545 (10.9)	148 (1.0)	11 (0.1)	0 (0.0)	1 (0.0)

AEs after IDA 12% versus 12.1% after DA, adjusted odds ratio for IDA versus DA 1.15, 95% CI 0.87–1.52, *P* = 0.316). See Statistical Methods for details.

^a^This table does not include data for 17 persons with missing Mf values who were assessed for AEs after treatment.

Abbreviations: (−), negative results; (+), positive results; AE, adverse event; DA, double-drug therapy (diethylcarbamazine, albendazole); IDA, triple-drug therapy (ivermectin, diethylcarbamazine, albendazole); Mf, microfilaremia; SAE, serious AE.

AEs varied by country study site (Table 4 and Fig 3), but this variability did not correspond to site-specific infection rates shown in Table 1. The vast majority of AEs were mild (grade 1). Only 1.2% of AEs in females and 0.9% of AEs in males had severity grades of 2 or higher. AEs were also more common in adults than in children and more common in females by univariable analysis despite their lower infection prevalence (Fig 3).

**Table 4:** AEs by country.

Country	Number of People Treated and Assessed for AEs	Any AE *n* (%)	Grade 1 *n* (%)	Grade 2 *n* (%)	Grade 3 *n* (%)	Grade 4 *n* (%)	SAE *n* (%)
**Haiti**	5,761	812 (14.1)	736 (12.8)	60 (1.0)	13 (0.2)	0 (0.0)	3 (0.1)
**India**	8,807	651 (7.4)	608 (6.9)	42 (0.5)	1 (0.0)	0 (0.0)	0 (0.0)
**Indonesia**	3,799	254 (6.7)	237 (6.2)	15 (0.4)	2 (0.1)	0 (0.0)	0 (0.0)
**PNG**	4,400	839 (19.1)	732 (16.6)	107 (2.4)	0 (0.0)	0 (0.0)	0 (0.0)
**Fiji**	3,419	591 (17.3)	550 (16.1)	33 (1.0)	5 (0.1)	0 (0.0)	3 (0.1)
**Total**	26,186	3,147 (12.0)	2,863 (10.9)	257 (1.0)	21 (0.1)	0 (0.0)	6 (0.0)

Abbreviations: AE, adverse event; PNG, Papua New Guinea; SAE, serious AE.

A multivariable logistic regression analysis showed that country, infection status (Mf more than isolated filarial antigenemia), age ≥ 18 years, and female sex were significantly associated with AEs (Fig 3). In contrast, treatment regimen was not significantly associated with AEs (adjusted odds ratio and 95% CI for IDA versus DA: 1.15 [0.87–1.52]).

### Types of AEs observed by treatment regimen

**Fig 4:**
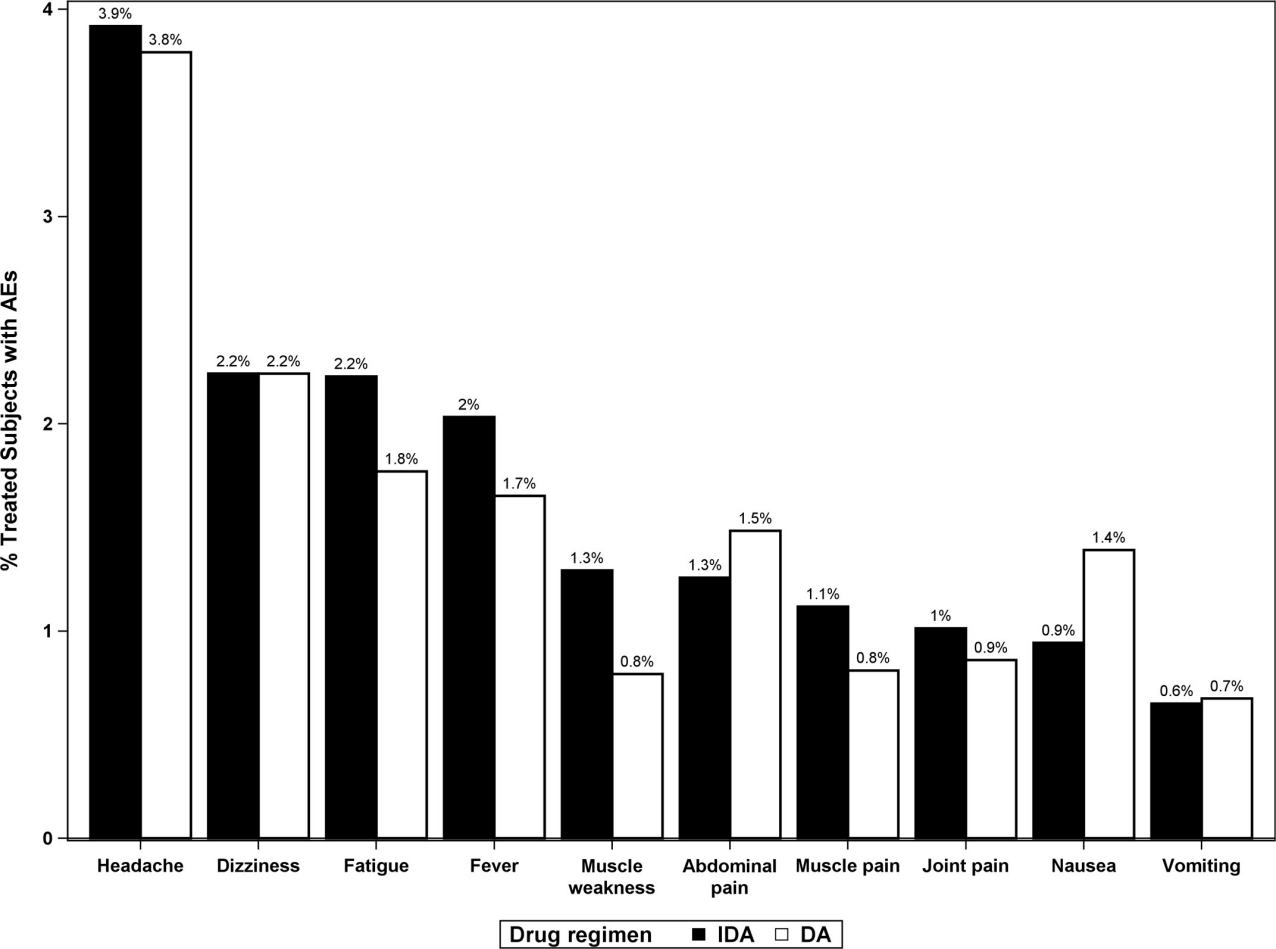
Frequencies of the most commonly observed AEs by treatment regimen expressed as percentages of participants who were assessed for AEs after treatment. In this analysis, participants can only count once for each AE type (e.g., a subject can only have a single headache). However, participants may have multiple different AE types (e.g., if a subject experiences a headache and fatigue, then they will be included in the numerator for both of these AE categories). AE, adverse event; DA, double-drug therapy (diethylcarbamazine, albendazole); IDA, triple-drug therapy (ivermectin, diethylcarbamazine, albendazole).

A total of 3,147 study participants (12% of the total number of participants assessed) reported at least one posttreatment AE (Table 4). The type, frequency, and severity of all AEs are listed in S3 Table. The most common AEs observed were headache, dizziness, fever, muscle aches, fatigue, and gastrointestinal problems, as expected. The types and proportions of AEs observed were similar after DA and IDA (Fig 4). AEs relating to the scrotum are of special interest when considering a new treatment regimen for LF, because these sometimes follow death of adult filarial worms in scrotal lymphatic vessels. Relatively few men in the study reported scrotal pain or swelling after treatment (51/12,912, 0.39%). Scrotal AEs were more common in men with Mf (1.9% versus 0.3%, *P* < 0.001) and more common after DA than after IDA (0.6% versus 0.2%, *P* < 0.001). Most scrotal AEs were of severity grade 1 (47 of 51, 92.2%). There were three grade 2 scrotal AEs (two after DA and one after IDA) and one grade 3 AE (after DA treatment). Other types of localized posttreatment AEs such as new onset or increased lymphedema, inguinal pain, or adenopathy were rarely observed (none with frequency > 0.1%, see S3 Table).

### Serious adverse events

SAEs after DA and IDA treatment were 0.04% (95% CI 0.01%–0.1%) and 0.01% (95% CI 0.00%–0.04%), respectively. Six SAEs were reported, and these are described in S3 Appendix. Five of these occurred after DA treatment, and one occurred after IDA. Five of six participants with SAEs were hospitalized for at least 1 night. The SAEs varied in severity, and none had a severity grade higher than 3. SAEs were only reported from Haiti and Fiji. Five of the SAEs were considered by attending physicians and medical monitors to have been possibly related to treatment, and one (abdominal pain in a 12-year-old boy after DA in Haiti) was considered to have been definitely related to treatment.

Three study participants were inadvertently treated during early pregnancy. A participant in Haiti had an uneventful pregnancy that resulted in the birth of a healthy baby girl. A participant in Fiji developed hyperglycemia during her pregnancy; she delivered a normal baby 42 weeks after her last menstrual period. A second participant in Fiji developed morning sickness during her pregnancy, as she had in previous pregnancies, and delivered a healthy baby at term.

## Discussion

This open-label, cluster-randomized study was conducted to assess the safety of a new triple-drug treatment regimen for use in LF elimination programs. When we designed this study, we had preliminary results from three small clinical trials that showed that IDA was much more effective than the widely used two-drug DA or IA regimens for achieving sustained clearance of Mf from the blood [7,8,9]. For example, a study in Papua New Guinea documented complete clearance of Mf from the blood in 96% of subjects 12 months after a single dose of IDA versus 25% with Mf clearance after DA [9]. A second study performed in Cote d’Ivoire documented complete Mf clearance in 76% of participants 1 year after IDA versus 26% with complete Mf clearance after IA [8]. Many subjects experienced AEs in these trials (59% after IDA versus 41% after DA in Papua New Guinea and 38% after IDA versus 39% after IA in Cote d’Ivoire). Moderate (grade 2) AEs were more common after IDA than after DA (27% versus 5%) or IA (19% versus 2%) in these studies, but no serious posttreatment AEs were observed in either study. Thus, results from these small studies suggested that the improved efficacy of IDA was accompanied by more frequent mild or moderate AEs. More safety data were needed before IDA could be recommended for widespread use in community settings.

This large, multicenter study was designed to fulfill requirements for policy changes regarding MDA for LF elimination at WHO. Results from our study show that MDA with the triple-drug IDA regimen was well tolerated in a variety of LF-endemic settings and generally as safe as the reference two-drug DA regimen. Although AEs were common after either treatment (and much more common in persons with microfilaremia), there was no excess of severe AEs or SAEs after IDA. High AE frequencies observed in clinical trials that required high Mf counts for inclusion were not observed in these community studies of populations with low to moderate infection prevalence. The lower frequencies of AEs observed after both IDA and DA in the community studies are highly relevant for the MDA use case.

The study was conducted in varied LF-endemic settings in five countries. Medical teams provided directly observed treatment and returned to assess the frequency and severity of posttreatment AEs according to uniform AE classification and scoring criteria. Overall, AE types and rates were similar after DA and IDA, and there were no significant differences by treatment regimen in rates of severe AEs or SAEs. SAE rates were below 0.1% after both treatments, and the low rate after IDA treatment (0.01%) satisfies one of WHO’s requirements for policy change.

This was one of the largest and most detailed studies to date of AEs assessed by active follow-up after MDA for LF. The study complied with recently published recommendations for reporting AEs in filariasis studies [11]. It is difficult to directly compare AEs in this study with prior studies as recently reviewed [11], because of differences in protocols for assessment. However, the frequencies and types of AEs observed in our study (mostly headache, fever, myalgia, dizziness, and fatigue) were well within ranges previously reported from community LF treatment studies. Most AEs were of mild to moderate severity (grades 1 or 2). Five of six SAEs reported in the study followed DA treatment. These results suggest that IDA should be as well tolerated as DA for use in MDA programs to eliminate LF.

One of the strengths of our study is that it was performed in a variety of settings in five countries across three WHO regions. This increases the generalizability of our results. Three of the five study areas had persistent LF despite seven or more prior rounds of MDA with DA, and two areas were treatment-naïve. Four study areas were endemic for *W*. *bancrofti* only, and one area was coendemic for *B*. *timori* and *W*. *bancrofti*. More than 26,000 participants were enrolled, and AEs were actively assessed by medical teams that followed a single assessment protocol that was compliant with GCP guidelines [13]. The rate of follow-up for assessment of AEs was very high, and this increases our confidence in the AE results.

Our study has a number of limitations. One limitation is that it was open-label, and that could have affected AE reporting by participants or AE assessment by clinical teams. A second limitation is that the treatment regimens were allocated by locality rather by household or individual. This could introduce bias because of correlations (environment, infection prevalence, genetic background, religion, or local culture) within localities. The diversity of study locations described as a strength above is also a potential limitation of the study. Aggregation of AE data across study sites could obscure variability among study sites. Another limitation of this study was that microfilaremia was assessed with 60-μl-thick blood smears rather than by the more sensitive method of membrane filtration of 1 ml of venous blood.

We do not believe that the open-label nature of the study affected AE reporting by participants or AE assessments by clinical teams, despite the larger number of tablets required for IDA treatment. Treatment regimen was randomly assigned by locality, because it would not have been feasible for us to conduct this study with double masking or with randomization of treatment allocation by individual. Our statistical analysis treated locality as a random effect to account for correlation among subjects within a locality. Although aggregation of AE results from five study sites obscures site-specific differences, we have provided an analysis of the country effect and presented some country-specific results in this paper to illustrate the range of AE frequencies and severity reported; more details will be provided later in country-specific publications. Some of the variability in AE rates by country may be related to cultural differences in the willingness of participants to report AEs, differences in LF prevalence, differences in prior use of MDA, or differences in comorbidities. However, this variability may also have been due to site-specific differences in interpretation and performance of AE assessments by study personnel despite the use of a common protocol and training materials across all five study sites. Venipuncture required for membrane filtration was not feasible for a study on this scale, and higher sensitivity for detection of Mf in persons with low blood Mf counts would not have altered conclusions about the relative safety of DA and IDA treatment.

As expected, AEs were significantly more common in persons with microfilaremia [11]. Among microfilaremic participants, persons who experienced AEs had higher mean Mf counts than persons who did not experience AEs. Filarial antigenemia was also associated with a slightly increased risk of AEs in amicrofilaremic persons. It is likely that some people in this group had low-level microfilaremia below the detection limit of 60-μl-thick blood smears. AE rates in persons with microfilaremia were significantly more frequent after IDA than after DA, and this may be due to more rapid clearance of Mf after IDA. However, there was no increased risk of severe or serious AEs in microfilaremic subjects after IDA treatment. Female sex, age ≥ 18 years, and study site (country) were all significant risk factors for AEs by both univariable and multivariable analyses. The sex effect was surprising, because males had significantly higher infection rates than females in each of the study sites. Since most of the excess AEs in females were of severity grade 1, it is possible that females were more likely than males to report mild AEs. Other studies have reported increased AE rates in females following MDA [21,22].

Although infection rates in adults were higher than in children, results of the multivariable analysis suggest that age was an independent risk factor for AEs following treatment. It was surprising that country/study site was also an independent risk factor for AEs; indeed, the India study site with a high infection rate had one of the lowest AE rates in the study. AE rates were not associated with the history of prior MDA in study sites. The low rate of posttreatment AEs in Indonesia is interesting, because the predominant filarial species present in those study sites was *B*. *timori*. Thus, it is reassuring that posttreatment AEs were not more common in areas with brugian filariasis. Comorbidities may partially explain the higher AE rates observed in the Papua New Guinea and Haiti study sites that had moderate or low microfilaremia rates, respectively. For example, the Papua New Guinea study area is highly endemic for malaria, and the study site in Haiti experienced flooding that may have resulted in stress or high rates of waterborne illnesses around the time of the safety study.

Results from this study have affected global health policy regarding LF elimination. WHO convened an independent Guidelines Development Group of experts in May 2017 to consider preliminary safety results from this study together with safety and efficacy data from randomized clinical trials of IDA for treatment of LF. New WHO guidelines published in November 2017 recommend use of IDA for LF elimination programs in endemic areas without coendemic onchocerciasis or loiasis that are not on track to eliminate LF by 2020 [23]. The reason for the geographic restriction is that DEC can cause SAEs in persons with onchocerciasis or loiasis. WHO’s announcement also emphasized the importance of coupling the introduction of IDA with programmatic changes designed to improve MDA compliance. Following that announcement, Merck & Co. announced a major expansion of their donation program to provide ivermectin for expanded use in MDA programs according to the new WHO guidelines [24].

In conclusion, results from this large open-label safety study show that MDA with the triple-drug IDA regimen was well tolerated in a variety of LF-endemic settings and generally as safe as the reference two-drug DA regimen. WHO now recommends use of IDA for LF elimination in certain settings (especially in countries that have used DA in the past and that are unlikely to meet elimination targets by 2020) [23]. This change in policy should facilitate large-scale rollout of IDA to facilitate elimination of this important neglected tropical disease.

## Supporting information

S1 CONSORT checklist (DOCX)

S1 Appendix A representative study protocol from one of the study sites (Haiti). (PDF)

S2 Appendix Statistical analysis plan. (PDF)

S3 Appendix Brief clinical summaries of serious adverse events reported after treatment. (DOCX)

S1 Table Adverse event scoring table. (DOCX)

S2 Table Rates of follow-up for assessment of adverse events after treatment. (DOCX)

S3 Table Detailed list of adverse events observed after treatment, by treatment group. (DOCX)

## Abbreviations

AE: adverse event
CFA: circulating filarial antigenemia
CI: confidence interval
CONSORT: Consolidated Standards of Reporting Trials
CRF: case report form
CTCAE: Common Terminology Criteria for Adverse Events
DA: diethylcarbamazine plus albendazole
DEC: diethylcarbamazine
DSMB: data safety monitoring board
EDC: electronic data capture
FAS: full analysis set
FTS: Filariasis Test Strips
FWA: Federal-Wide Assurance
GCP: good clinical practice
GLMM: generalized linear mixed model
GPELF: Global Programme to Eliminate Lymphatic Filariasis
IA: ivermectin plus albendazole
ICH GCP: International Council for Harmonization on Good Clinical Practice
IDA: ivermectin with diethylcarbamazine and albendazole
IRB: institutional review board
LF: lymphatic filariasis
MDA: mass drug administration
Mf: microfilariae
MSD: Merck Sharp & Dohme
SAE: serious AE
WHO: World Health Organization
