# Supplementary material for: The safety of double- and triple-drug community mass drug administration for lymphatic filariasis: A multicenter, open-label, cluster-randomized study
Source: PLoS Med. 2019 Jun 24;16(6):e1002839. doi: 10.1371/journal.pmed.1002839 (PMC6590784; doi:10.1371/journal.pmed.1002839)
Supplement: S1 Appendix — (PDF) [file pmed.1002839.s002.pdf]

“Community Based Safety Study of 2-drug (Diethylcarbamazine and Albendazole) versus 3-drug (Ivermectin, Diethylcarbamazine and Albendazole) Therapy for Lymphatic Filariasis in Haiti”

Protocol Identifier: DOLF_IDA_Haiti

Type: Community-Based Mass Drug Administration

Funding Sources: This project is funded by the Bill and Melinda Gates Foundation through the Death for Onchocerciasis and Lymphatic Filariasis Project (DOLF), Washington University in St. Louis.

Project Principal Investigator(s): Gary Weil, MD, Washington University, USA
Christopher King, MD, PhD, MPH, Case Western Reserve University, USA

Principal Investigator for MSPP: Jean Frantz Lemoine, MD, MPH
Ministère de la Santé Publique et de la Population (MSPP), Haïti

Principal Investigator for CDC: Christine Dubray, MD, MSc
Centers for Disease Control and Prevention (CDC), Atlanta, GA, USA

CDC Co-Investigators: LeAnne Fox, MD, MPH**;** Anita Sircar, MD, MPH**;** Kimberly Won**;** Ryan Wiegand, PhD

Non-CDC Co-Investigators: Valery D. Madsen Beau de Rochars, MD, MPH ; University of Florida (UF)**;** Abdel N. Direny, MD, MPH; RTI International (RTI)**;** Carl Renand Fayette, MD; IMA World Health (IMA), Port au Prince, Haiti and Washington, DC

Initial Protocol: v.1.0 24 March 2016

Amendment 1: v.2.0 22 May 2016

Amendment 2: v.2.1 22 August 2016

INVESTIGATOR AGREEMENT

"Community Based Safety Study of 2-drug (Diethylcarbamazine and Albendazole) versus 3-drug (Ivermectin, Diethylcarbamazine and Albendazole) Therapy for Lymphatic Filariasis in Haiti"

DOLF_IDA_Haiti: v2.1   22 August 2016

I have read the protocol, including the appendices, and I agree that it contains all necessary details for me and my staff to conduct this study as described. I will conduct this study as outlined and make a reasonable effort to complete the study within the time designated.

I will provide all study personnel, participating in the study under my supervision copies of the protocol and access to all study related information provided by the DOLF project. I will discuss with them to ensure they are full informed about the study drug(s) and the study procedures.

Principle Investigator: ____

Name/Title (Print/Type)

Signed: ____ **Date:** _________________

NOTE: Both the Project PI and local PI should have signed investigator agreements on file.

TABLE OF CONTENTS

INVESTIGATOR AGREEMENT 2
TABLE OF CONTENTS 4
PRINCIPAUX ROLES ET RESPONSABILITÉES 7
LIST OF ABBREVIATIONS 9
1 PROTOCOL SUMMARY 10
2 BACKGROUND INFORMATION AND RATIONALE 14
2.1 General Context 14
2.2 Country Specific Background 14
3 POTENTIAL RISKS AND BENEFITS 17
3.1 Risks of Blood Draw 17
3.2 Risks of Study Drugs 17
3.3 Potential Participant and Community Benefit 17
3.4 Participant Participation and Cost 18
3.5 Compensation for Injury 18
4 STUDY DESIGN AND OBJECTIVES 18
4.1 Study Objectives 18
4.1.1 Primary objective 18
4.1.2 Secondary Objectives 18
4.2 Study Design 19
4.3 Study Screening and Enrollment 19
4.4 Study Sites 19
4.5 Preparatory Activities 19
4.5.1 Social Mobilization 19
4.5.2 Household Enumeration, Census and Geo-Referencing 20
4.6 Pre-Treatment Assessment Team 20
4.6.1 Inclusion and Exclusion Criteria 20
4.6.2 Pregnant Females 21
4.6.3 Informed Consent 21
4.6.4 Baseline Survey 21
4.6.5 Screening for Filarial Antigenemia and Microfilaria 22
4.6.6 Randomization 22
4.7 Withdrawal 22
4.8 Efficacy and Effectiveness of IDA vs DA 22
4.9 Retreatment 23
4.10 Guidelines for Stopping the Trial 23
4.11 Triple Drug Regimen Acceptability 23
5 INVESTIGATIONAL PRODUCT 24
5.1 Study Drug Background 24
5.2 Product Supply and Storage 25

5.3 Triple Drug Therapy (IDA) and Two-Drug Therapy (DA) 25
6 SAFETY REPORTING AND SAFETY MONITORING 25
6.1 Definitions..25
6.2 Assessment of Adverse Events 26
6.3 Serious Adverse Event (SAE) Assessment and Management 27
6.4 Reporting of Pregnancy 27
6.5 Safety Monitoring by the Oversight Committee 27
6.6 Country Specific Safety Reporting 28
7 CLINICAL MANAGEMENT OF EVENTS 28
7.1 Adverse Event Monitoring and Management 28
7.1.1 Mild Localized Symptoms...28
7.1.2 Moderate to Severe Localized Adverse Events 29
7.1.3 Moderate Systematic Adverse Events...29
7.1.4 Moderate to Severe Systemic Adverse Events..29
7.2 Rapid Response Teams for Management of Adverse Events 29
8 STATISTICAL CONSIDERATIONS 29
8.1 Safety 29
8.2 Efficacy...30
8.3 Enrolling Additional Participants...30
9 DATA HANDLING/RECORD KEEPING/SOURCE DOCUMENTS 31
9.1 Types of Data Collected...31
9.2 Study Records Retention 32
9.3 Source Documents 32
10 RESPONSIBILITIES 32
10.1 Investigator Responsibilities..32
10.2 Good Clinical Practice..32
10.3 Institutional Review Board (IRB)/Ethics Committee (EC) 32
10.4 Informed Consent 33
10.4.1 Informed Consent Training 33
10.4.2 Country Specific ICF Information..33
10.5 Participant Privacy 34
10.6 Data Ownership 34
11 PUBLICATION POLICY 35
12 LITERATURE REFERENCES 36
13 LIST OF APPENDICES.. 37
Appendix 1: Study Flow Diagram (Country Specific) 38
Appendix 2: Participant Enrollment Form [Example] 39
Appendix 3: Participant Monitoring Form [Example].. 42
Appendix 4: Guide to Assigning Adverse Event Severity..................................... 45
Appendix 5: Adverse Event Evaluation and Report Form (AEERF) [Example].... 48
Appendix 5a: Required Reporting Guideline For Serious Adverse Events 52
Appendix 6: Information Sheet and Informed Consent Form [Example].............. 56

PRINCIPAUX ROLES ET RESPONSABILITÉES

**Principal Investigator for MSSP:**	Jean Frantz Lemoine, MD, MPH<br>Programme National de la Malaria et de la Filariose Lymphatique (PNCM)<br>Ministère de la Santé Publique et de la Population (MSPP)<br>Address: Rue Monseigneur Guilloux,<br>Palais des Ministères, Port-au-Prince, Haïti.<br>Phone number: +1 (509) 3744-8755 or +1 (509) 3946-3026)<br>Email: tileum@hotmail.com
**Principal Investigator for CDC:**	Christine Dubray, MD, MSc<br>Division of Parasitic Diseases and Malaria, CDC<br>Address: 1600 Clifton Road, MS A-06<br>Atlanta, GA 30317, USA<br>Phone number: +1(404)718-4769<br>Email: ffg5@cdc.gov
**Co-Investigators:**	LeAnne Fox, MD, CDC<br>Phone number: +1(404)718-4739<br>Email: llf4@cdc.gov<br>Anita Sircar, MD, MPH, CDC<br>Phone number: +1(404)718-4728<br>Email: yxi6@cdc.gov<br>Kimberly Won, CDC<br>Phone number: +1(404)718-4137<br>Email: kfw7@cdc.gov<br>Valery .Madsen Beau de Rochars, MD, MPH<br>Emerging Pathogens Institute, University of Florida<br>Phone number: (352) 294-5695<br>Fax: (352) 273-9430<br>Email: madsenbeau@phhp.ufl.edu<br>Abdel N. Direny, MD, MPH, RTI<br>Phone number: +1 (202) 974-7814<br>Email: adireny@rti.org<br>Carl Renand Fayette, MD, IMA
**Protocol Statistician:**	Ryan Wiegand, PhD, CDC<br>Phone number: +1(404)639-2031<br>Email: fwk2@cdc.gov

<u>Roles and Responsibilities of Partners:</u>

- MSPP: MSPP is responsible for developing the protocol, submitting the protocol to the National Ethical Committee in Haiti, training study personnel on study methodology,

including individuals in the study, collecting, managing and analysing data, and preparing manuscript and abstract.

- CDC: CDC will provide technical assistance to MSPP, train study personnel on study methodology, and assist with data analysis and manuscript preparation. CDC personnel will not intervene or interact with living individuals included in the study or have access to personally identifiable information for research purposes.
- UF and RTI: UF and RTI personnel will act as consultants to provide technical assistance to MSPP to assist with training of study personnel, appropriate implementation of standard operating procedure during the study, analysis of data, and preparation of manuscript.
- IMA: IMA will act as the implementing partner for MSPP and will assist with study logistics, finance, hiring study personnel, and organization of trainings.

LIST OF ABBREVIATIONS

GENERAL PROJECT ABBREVIATIONS

AE	Adverse Event/Adverse Experience
AEERF	Adverse Event Evaluation and Report Form
ALB	Albendazole
CDC	Centers for Disease and Control and Prevention
CDD	Community Drug Distributor
CRF	Case Report Form
DA	Two Drug Therapy (dyethilcarbamzine and albendazole)
DEC	Diethylcarbamazine
DOLF	Death for Onchocerciasis and Lymphatic Filariasis
DOT	Directly Observed Treatment
DSMB, DSRB or DMC	Data and Safety Monitoring Board also called Data Safety Review Board or Data Monitoring Committee
EC	Ethics Committee (may also be called IRB or Institutional Review Board)
EDC	Electronic Data Capture
FTS	Filariasis Test Strip
GCP	Good Clinical Practice
GPELF	Global Programme to Eliminate Lymphatic Filariasis
GPS	Global Positioning System
ICF	Informed Consent Form
ICH	International Conference on Harmonization
ICT	Immunochromatographic Test
IDA	Triple Drug Therapy (Ivermectin, Diethylcarbamazine, and Albendazole)
IMA	IMA World Health

IRB	Institutional Review Board (may also be called EC)
IVM	Ivermectin
LF	Lymphatic Filariasis
MDA	Mass Drug Administration
MF	Microfilaria(e)
Mg	Milligram
NTD	Neglected Tropical Diseases
NLM	National Library of Medicine
PI	Principal Investigator
SAE	Serious Adverse Event/Serious Adverse Experience
SOP	Standard Operating Procedure
TAS	Transmission Assessment Surveys
UNID	Unique Study Identification Numbers
USAID	United States Agency for International Development
WHO	World Health Organization

COUNTRY SPECIFIC ABBREVIATIONS

MSPP	Ministère de la Santé Publique et de la Population

1 PROTOCOL SUMMARY

**Study Title:**	Community Based Safety Study of 2-drug (Diethylcarbamazine and Albendazole) versus 3-drug (Ivermectin, Diethylcarbamazine and Albendazole) Therapy for Lymphatic Filariasis in Haiti
**Type of Study:**	Mass Drug Administration
**Population:**	IDA/ Triple Drug Arm: participants more than or equal to 5 years of age<br>DA/ Dual Drug Arm (DA): participants more than or equal to 5

	years of age
**Number of Treated Areas:**	Commune of Quartier Morin
**Duration of Study Participant Participation:**	Single treatment with daily adverse event follow-up thru Day 7, then a long-term follow-up at 1 year.
**Study Drugs**	Ivermectin (3 mg tablets) **not included in two arm treatment*<br>Diethylcarbamazine (100 mg tablets)<br>Albendazole (400 mg tablets)
**Primary Objectives:**	Determine the frequency, type, and severity of adverse events following triple drug therapy (IVM+DEC+ALB) compared to the standard two drug treatment (DEC+ALB) in infected and uninfected individuals in a community
**Secondary Objectives:**	Compare the efficacy of IDA (3 drug therapy) to DA (2 drug therapy) administered in communities for clearance of MF and filarial antigenemia<br>Assess the effect of intensity of filarial infection on the frequency and severity of adverse events<br>Compare community acceptance of Mass Drug Administration with three drug vs two drug therapy
**DOLF Project Integration**	This protocol is specific to Haiti, but the results will also be included in the larger inter-country DOLF project. Data will be available/reviewed at a country level and at the project level.

In 2000, the World Health Organization (WHO) launched the Global Programme to Eliminate Lymphatic Filariasis (GPELF) to eliminate lymphatic filariasis as a public health problem by 2020. To interrupt transmission, WHO recommends therapy using combinations of two medicines delivered to entire at risk populations through a strategy known as mass drug administration. Ivermectin and albendazole are administered in areas where onchocerciasis is co-endemic; diethylcarbamazine and albendazole are administered in areas where onchocerciasis is not co-endemic.

Results of a pilot study in Papua New Guinea suggest that triple drug therapy (ivermectin, diethylcarbamazine and albendazole) is superior to the currently recommended two-drug regimen. A single dose of the triple therapy rapidly achieved complete clearance of *Wuchereria bancrofti* microfilariae from the blood of 12 individuals for at least one year post-treatment. All six individuals tested at 24 months were still amicrofilaremic, suggesting that the triple therapy might permanently sterilizes adult filarial worms. Many people treated in these studies experienced transient systemic adverse events commonly associated with diethylcarbamazine or ivermectin treatment of filariasis. Adverse events were more frequent after the triple therapy than after the usual combination of two drugs. However, no serious adverse events were observed. The dramatic reduction and sustained decrease of microfilaria along with the safety profile seen in the Papua New Guinea studies suggest that the triple drug therapy may be a useful tool to achieve the goal of eliminating lymphatic filariasis as a public health problem by 2020.

Although the study cited above has clearly demonstrated the superiority of the triple therapy for clearing *W. bancrofti* microfilaria from the blood, more safety and efficacy data are needed before triple therapy can be rolled out on a large scale as a mass drug administration regimen in lymphatic filariasis endemic countries. WHO recommends a best practice called “cohort event monitoring” for demonstrating safety of new drug regimens for public health program use. Establishing safety through such methodology requires pre and post treatment assessment from at least 10,000 people treated with the triple therapy across multiple settings.

It is therefore proposed to conduct a cohort event monitoring study to acquire safety data in Haiti (Quartier Morin Commune). Efficacy and acceptability components will also be included in the study. Similar studies will be conducted simultaneously in India, Indonesia, Papua New Guinea, Fiji and Sri Lanka to reach the 10,000 people necessary to assess the safety of this new drug combination.

STUDY DESIGN

General Flow Diagram:

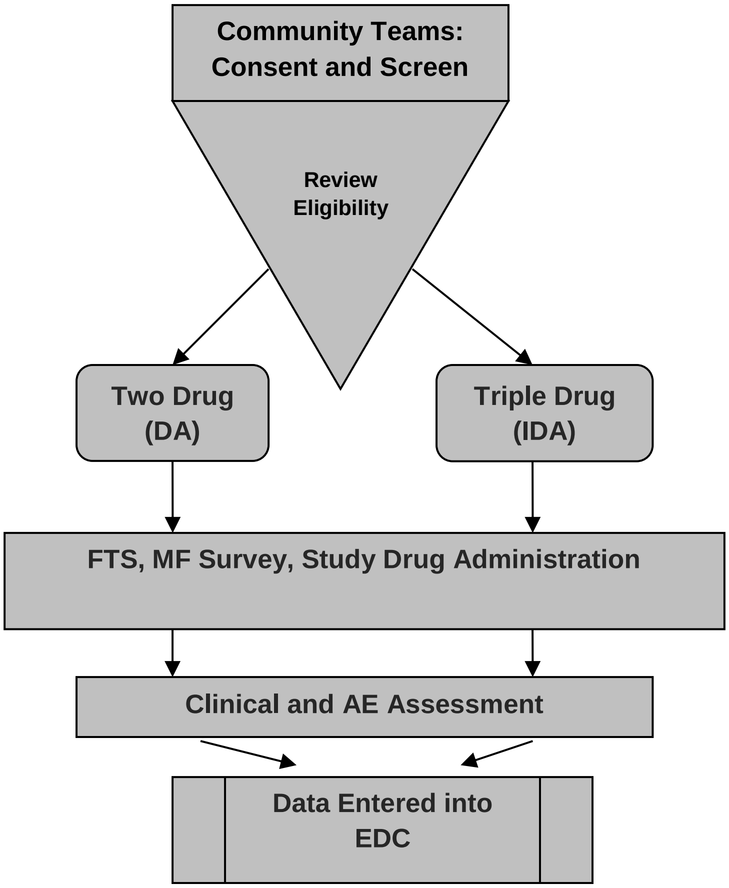

DOLF_IDA_Haiti Study	ARM 1	Sample Size: 3000	Triple Drug
	ARM 2	Sample Size: 3000	Two Drug

NOTE: A Study Flow Diagram specific for Haiti is provided in Appendix 1.

2 BACKGROUND INFORMATION AND RATIONALE

2.1 General Context

In 2000, the World Health Organization (WHO) launched the Global Programme to Eliminate Lymphatic Filariasis (GPELF) to eliminate lymphatic filariasis as a public health problem by 2020 [1] To interrupt transmission, WHO recommends therapy using combinations of two medicines delivered to entire at-risk populations through a strategy known as mass drug administration (MDA). Ivermectin and albendazole are administered in areas where onchocerciasis is co-endemic; diethylcarbamazine and albendazole are administered in areas where onchocerciasis is not co-endemic.

Results of a pilot study in Papua New Guinea suggest that triple drug therapy (ivermectin, diethylcarbamazine and albendazole) [2] is superior to the currently recommended two-drug regimen) [3]. A single dose of the triple therapy rapidly achieved complete clearance of *Wuchereria bancrofti* microfilariae from the blood of 12 individuals for at least one year post-treatment. All six individuals tested at 24 months were still amicrofilaremic, suggesting that the triple therapy might permanently sterilizes adult filarial worms. Many people treated in these studies experienced transient systemic adverse events commonly associated with diethylcarbamazine or ivermectin treatment of filariasis. Adverse events were more frequent after the triple therapy than after the usual combination of two drugs. However, no serious adverse events were observed. Preliminary results from two larger clinical trials in Papua New Guinea and in Cote d'Ivoire (West Africa) are consistent with results from the pilot study. The dramatic reduction and sustained clearance of microfilaremia along with the safety profiles seen in these studies suggest that the triple drug therapy may be a useful tool for achieving the goal of eliminating lymphatic filariasis as a public health problem by 2020.

Although the studies mentioned above have clearly demonstrated the superiority of the triple drug therapy for clearing *W. bancrofti* microfilariae from the blood, more safety and efficacy data are needed before triple therapy can be rolled out on a large scale as a mass drug administration regimen in lymphatic filariasis endemic countries. WHO recommends a best practice called "cohort event monitoring" for demonstrating safety of new drug regimens for public health program use. Establishing safety through such methodology requires pre and post treatment assessments from at least 10,000 people treated with the triple therapy across multiple settings.

2.2 Country Specific Background

Haiti is one of only four countries in the Americas where transmission of LF still occurs [4]. In 2001, a national survey undertaken by the Ministère de la Santé Publique et de la Population (MSPP) and partners was able to show that approximately 90% of the Haiti's 140 communes (district level) needed mass drug administration (MDA), representing an at-risk population of nearly 8 million people. Figure 1 shows the results of the 2001 LF mapping by commune divided between colored zones. The red zone or zone rouge represents the areas with highest

transmission. Following the completion of mapping, the MSPP established three goals for the National Program to Eliminate LF: (1) to interrupt transmission, (2) to reduce the suffering of persons with clinical and chronic manifestations of LF, and (3) to encourage positive health behaviors [5]. The MSPP National Neglected Tropical Diseases (NTD) Strategic Plan specifies that the goal is to eliminate LF in Haiti by 2020. Haiti follows the LF strategy for elimination through consecutive MDAs with diethylcarbamazine (DEC) and albendazole for at least five to six years [6]. Even though LF was not identified in every commune, all communes were targeted for MDA. In 2000, MDA first started in Léogâne under the umbrella of a CDC-funded demonstration project [7]. In 2012, MSPP achieved 100% geographic coverage. Because of a gap in funding, national coverage did not continue in 2013, when 13 communes were untreated. In 2014 and 2015, MSPP was once again able to reach 100% geographic coverage with funding and technical support from its partners USAID (through the ENVISION project), the University of Notre Dame, and the Centers for Disease and Control and Prevention (CDC).

Figure 1: **Lymphatic Filariasis Baseline Mapping by Commune in Haiti, 2001**

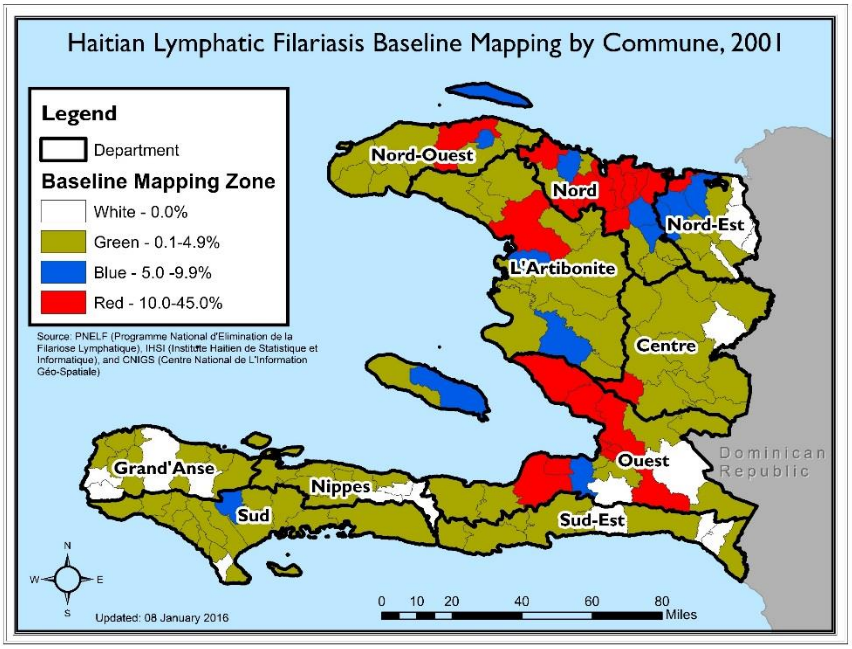

Haiti has made significant progress in reaching its program goals. In 2015, MSPP carried out LF transmission assessment surveys (TAS) in 48 of Haiti's 140 communes, with 46 of them passing the TAS successfully. Only four communes did not pass the pre-TAS, which aimed at demonstrating that the ICT prevalence in a commune is <2% and that the commune is eligible to conduct the TAS. Five high-prevalence communes (zones rouges, Figure 1) have passed the TAS, which is remarkable given their high starting prevalence (14%–45%). Only two communes in the North Department (Limonade and Plaine-du-Nord) being part of the zone rouge failed the TAS in 2015, while another (Cap-Haïtien) passed marginally [8]. MDA will continue in these

communes and in the communes that did not pass the pre-TAS for a minimum of two years. Figure 2 represents the LF status by commune in 2016.

Figure 2: Lymphatic Filariasis Program Status by Commune in Haiti, 2016

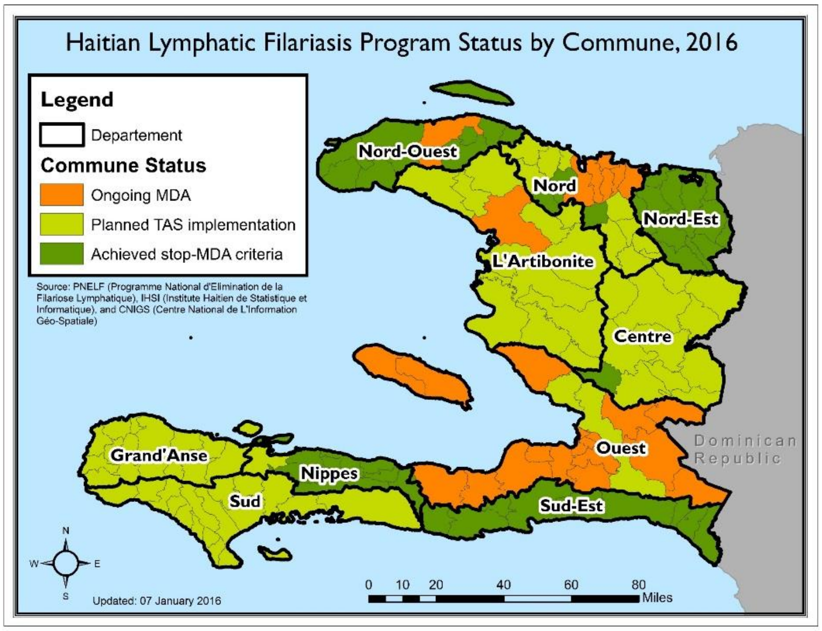

Establishing safety for MDA through WHO defined “cohort event monitoring” requires pre- and post-treatment assessment from at least 10,000 people treated with IDA across multiple settings (3,000 from Haiti). The current two-drug MDA regimens were studied in closely monitored community trials in a similar manner before they were endorsed for widespread use in the GPELF. It is therefore proposed to conduct a cohort event monitoring study to acquire similar safety data in Haiti (Quartier Morin Commune) before the new IDA regimen can be used to accelerate LF elimination particularly in those communes where LF antigenemia remains ≥1%.

3 POTENTIAL RISKS AND BENEFITS

3.1 Risks of Blood Draw

Blood collection via finger prick is considered to be minimal risk and little or no discomfort is anticipated. The risk of infection is minimized by the use of standard sterile techniques. On occasion a participant may faint during or after the finger prick. Study personnel will be alert to participant reactions after the blood collection and will provide aid as needed

3.2 Risks of Study Drugs

The combinations of ivermectin plus albendazole or DEC plus albendazole are widely used for MDA. There have also been clinical trials of DEC plus Ivermectin and for triple drug therapy that show no significant drug interactions. Risks of each study drug is summarized below:

Diethylcarbamazine (DEC): The most common side effects reported are itching and swelling of face, headache, joint pain, unusual tiredness or weakness. These are transient. Less common are dizziness, nausea or vomiting. Fever, painful and tender glands in groin, neck and armpits or skin rash can occur, and are usually associated with high burdens of infection as judged by the level of blood microfilaremia.

Albendazole (ALB): The most common side effects reported are headache, nausea, stomach pain and vomiting and are usually associated with heavy soil-transmitted helminths infections. Severe allergic reactions occur rarely, and include rash, hives, itching, difficulty breathing, tightness in the chest, swelling of the mouth, face, lips, or tongue, and dark urine. Mild elevation in liver transaminases can occur, but normalize with cessation of treatment. These AEs are usually associated with prolonged ALB therapy.

Ivermectin (IVM): The most common side effects reported are diarrhea, dizziness and nausea. Rare side effects include rash, hives, itching, difficulty breathing, chest tightness, swelling of the mouth, face, lips, or tongue, eye pain, fainting, and fast heartbeat. Mild decrease in leukocyte counts, elevated liver function tests, and cardiovascular effects that included tachycardia and orthostatic hypotension have been described. Infrequently, treatment can exacerbate bronchial asthma. These AEs are usually associated with prolonged therapy.

3.3 Potential Participant and Community Benefit

Infected individuals, who sign an informed consent, will be treated for the LF infection. LF transmission to the community will be reduced by participation in either treatment arm. A broader community benefit may be facilitated by the triple drug regimen as it is believed the triple drug regimen has the potential to markedly reduce the number of MDA treatments needed to achieve transmission interruption and elimination of LF.

Both regimens provide treatment for intestinal worms, and the triple drug treatment has the added benefit of providing an effective treatment for scabies.

If the triple drug intervention proves successful, the triple therapy is likely to be adopted in many LF endemic areas globally. In order to facilitate such an uptake of triple therapy into national treatment policies, the study will be performed by the Coordinator of the LF and Malaria National Programs (MSPP), Dr. Jean Frantz Lemoine and results from this study will be combined and shared with the World Health Organization.

3.4 Participant Participation and Cost

Participation is voluntary and participants may decline participation without consequences. There will be no cost to the participant to participate in the study and they will not be paid for their participation. The study will cover cost associated with laboratory test, study drugs, and clinical monitoring.

3.5 Compensation for Injury

The study drugs have been widely used for treatment of LF and it is anticipated that injury resulting from treatment will be rare. In the event that a participant experiences a serious adverse event (SAE) attributable to study treatment, the project will help in supporting the medical treatment and/or hospitalization required.

4 STUDY DESIGN AND OBJECTIVES

4.1 Study Objectives

4.1.1 Primary objective

To determine the frequency, type and severity of adverse events following triple-drug therapy (IVM+DEC+ALB, IDA) compared to the standard two-drug treatment (DEC+ALB, DA) in infected and uninfected individuals in a community.

4.1.2 Secondary Objectives

1. To compare the efficacy of IDA vs. DA administered in communities for clearance of MF and filarial antigenemia.
2. To assess the effect of intensity of filarial infection on the frequency and severity of adverse events.
3. To compare community acceptance of MDA with IDA vs. DA.

4.2 Study Design

The trial will be an open labelled two-armed study. The two arms are (1) MDA with IDA (triple drug therapy) and (2) MDA with the currently used combination of DA (two-drug regimen). An overview of the study flow is provided in Appendix 1.

The primary endpoint will be the rate of AE and SAE among participants. The definitions of mild, moderate, severe and serious AE are provided in Appendix 4.

4.3 Study Screening and Enrollment

4.4 Study Sites

The study will be conducted in the commune of Quartier Morin in the Northern Department. The baseline ICT prevalence in the commune of Quartier Morin was 39% (2001). In 2014, seven rounds of consecutive MDA had been conducted. The same year, the commune failed the pre-TAS. The pre-TAS consists of collecting data in a sentinel site and in a spot check site at least six months after the fifth round of MDA to assess whether the TAS should be implemented. Ideally, the sites should collect data from 300–500 individuals aged over 5 years chosen from an area of known high transmission or from an area where difficulty in achieving high drug coverage is anticipated. The results of the assessment should show the prevalence of microfilaraemia to be <1% or the prevalence of ICT to be <2% in all sites in order to be eligible for the TAS. In the commune of Quartier Morin, the ICT prevalence found during the pre-TAS was 4.2% (21/496) of which of which 38% were MF positive (8 positive, 1.6% of all tested).

4.5 Preparatory Activities

4.5.1 Social Mobilization

Prior to the administration of the drugs, intense social mobilization activities will be conducted to ensure maximum community participation. This will include development and distribution of key messages that will emphasize the acceptance and swallowing of the drugs along with their benefits and safety.

Print and electronic mass media, people-based and folk media (e.g. sound trucks) will be used in the social mobilization campaign. Interpersonal communication using tool kits will play an important role utilizing the opportunity provided during enumeration and other preparatory activities.

4.5.2 Household Enumeration, Census and Geo-Referencing

Health workers with the research team and community drug distributors (CDD) will enumerate and record the GPS coordinates of each house and compound within the selected study areas (House Visit #1, Appendix 1). A census will be performed to collect name, age and sex of each household member. Basic information on house structure that might affect mosquito exposure to lymphatic filariasis infection, e.g. type of structure, whether screened windows present, existence of a toilet, running water, electricity and/or insecticide treated bed nets will also be collected.

4.6 Pre-Treatment Assessment Team

The pre-treatment assessment (House visit #2, Appendix 1) team will be composed of individuals with basic medical training able to perform a medical history and a basic physical examination (local health workers, physicians, and nursing or medical students), laboratory technicians, and community drug distributors involved in previous MDA for LF and known by the local community.

4.6.1 Inclusion and Exclusion Criteria

Inclusion Criteria

1. Age ≥ 5 years, for IDA and DA arms (males and females).
2. Able to provide informed consent or give parental consent for minors to participate in the trial
3. No evidence of severe or systemic co-morbidities except for features of filarial disease

Exclusion Criteria

1. Age < 5 years (ivermectin is not approved for use in children less than 5 years of age)
2. Unable to provide informed consent or give parental consent for minors to participate in the trial
3. Pregnant women (DEC, ivermectin and albendazole are not known to be safe for use during pregnancy)
4. Severe chronic illness (chronic renal insufficiency, severe chronic liver disease, or any illness that is severe enough to interfere with activities of daily living)
5. History of previous allergy to MDA drugs

4.6.2 Pregnant Females

Pregnant females will not be eligible to participate in this study because of the unknown effects of the drugs and drug combination used in this study. Females will be asked about the timing of the first day of their last menstrual period. Females who report that their last menstrual period started 4 weeks or longer before the interview will be excluded from the study. Females who do not recall the timing of their last menstrual period will also be excluded.

4.6.3 Informed Consent

All participants will provide written informed consent before any study procedures are done. Participation of minors (7−17 years of age) will require their assent and the written consent of at least one parent. Participants will sign a written, informed consent before the inclusion process (Appendix 6). In the event that a participant is unable to read or has insufficient level of knowledge to comprehend the consent form, another person (family member, neighbor or other community member) with sufficient reading and writing skills will act as a witness to the consenting process. The witness should not be involved in the implementation of the study. Before they give their consent, individuals will be evaluated to know if they meet the study inclusion/exclusion criteria. Copies of the information sheet and of the consent/assent form will be given to each participant. The phone number to call would an AE occurs will be at the bottom of the informed consent.

A waiver of consent is being requested for the Census and Geo-referencing portion of the study prior to receiving formal consent. The study team will be collecting information about the communities and residents. This portion of the study is not greater than minimal risk and the members of the study team who are conducting the Census and Geo-referencing portion of the study will explain what they are collecting to village residents.

4.6.4 Baseline Survey

After consenting and prior to evaluation for LF infection and treatment, all individuals will be assigned a unique ID and be enrolled using a participant enrollment form (Appendix 2). Questions will be asked to each participant about their general health and last menstrual period (to establish pregnancy for women of childbearing age). Each individual will be asked if they have signs of LF complications (hydrocele, lymphedema, lymphangitis, and lymphadenitis), if they took treatment during the previous MDA for LF and if they recently took albendazole, diethylcarbamazine, or ivermectin for other conditions. Participants reporting lymphedema will be examined to identify the location of the lymphedema. Participants will also be examined for scabies. Finally, participants will also be evaluated for the same AE than those evaluated after the treatment (Appendix 2).

4.6.5 Screening for Filarial Antigenemia and Microfilaria

Approximately 75µl of capillary blood from each eligible individual will be collected via finger prick to be deposited on the rapid diagnostic test Filariasis Test Strip (FTS, Alere™, WHO approved) for LF antigen detection in the field. Participants with positive FTS tests will be visited at night (10 - 12 am) for microfilaria testing (60 µl measured volume blood smear- 3 lines, prepared according to the project Standard Operating Procedure (SOP)) collected by the finger prick method.

Additionally, 60µl of capillary blood will be applied to a filter paper. The dried filter disks will be stored in a cool, dry place until used for testing. Study participants will be informed that their blood on filter paper will be tested for LF antibody and potentially for other diseases of public health importance and will be shipped to the United States and stored for a longer time after the intended testing. No HIV or genetic testing will be performed.

Universal precautions for individuals collecting and working with blood samples to include proper disposal of contaminated materials (test strips, lancets, capillary tubes, blood film slides) will be in accordance with the guidelines prescribed by the local health authorities.

4.6.6 Randomization

Communities will be assigned treatment either by randomization or by purposively matching communities based on population and prevalence of LF. If the prevalence is homogenous across the communities, each site may be randomly assigned to one of the two treatment arms. If the prevalence is heterogeneous, communities will be selected into each arm so that the population and prevalence between the two treatment arms is similar.

4.7 Withdrawal

Participation in this study is completely voluntary, and participants may terminate participation at any time. Also if the well-being of the participant is compromised in any way, based on the opinion of the investigator, the participant can also be withdrawn from the study. Even if the participant leaves the project early, we will encourage them to contact us at any time within the month after treatment to report any possible study-related AEs.

4.8 Efficacy and Effectiveness of IDA vs DA

One year post MDA, all participants who were positive for either microfilaremia or filarial antigenemia during the baseline visit will be tested for filarial antigen using the FTS to assess their response to treatment and to compare the efficacy of the two treatment regimens. Participants with positive FTS will also be tested for nocturnal microfilaremia by blood smear (finger prick).

4.9 Retreatment

Participants with persistent infection (antigenemia or microfilaremia) at 12 months will be re-treated with the standard MDA regimen (a single dose of DEC with Albendazole).

4.10 Guidelines for Stopping the Trial

There are no pre-specified criteria for terminating the study early.

Upon review of the data for the trial, the data and safety monitoring board (DSMB) will make decisions regarding the continuation of the trial. The final decision to stop the trial is left to the recommendation of the DSMB. If the DSMB recommends discontinuation or modification of the study, the Chair of the DSMB will meet or talk with the DOLF Project Team at the earliest opportunity to review the basis for the recommendation. The study should be stopped if a treatment arm shows a significant increase in unacceptable side effects that would include, death, fever, and nausea that persist more than a day and would require hospitalization.

4.11 Triple Drug Regimen Acceptability

A survey to assess the treatment acceptability in the community is planned to follow the safety trial. The overall aim is to understand the community's acceptance of the 3-drug regimen as well as gain insight into the feasibility of administering this new therapy in the future. Part of the investigation will include assessing community member's perception of the possible side effects experienced as a result of the 3-drug therapy compared to the 2-drug therapy, and how that might affect future rounds of mass drug administration (MDA) at the community level.

Community acceptance will be measured using a survey to community members receiving both the 2-drug and 3-drug treatments during the safety trial. The survey participants will be identified from the roster of individuals enrolled in the safety trial. To complement this survey, a series of focus group discussions in the community as well as key informant interviews are proposed with community leaders, health personnel and drug distributors in the same communities to assess perceptions about the 3-drug versus the 2-drug regimen. The community acceptability study will be carried out within one month of the completion of the safety trial. The protocol for the acceptability survey is included in of this protocol. The community questionnaire and topic guides will be submitted to the ethical committee for approval as an amendment prior to implementation of the survey.

5 INVESTIGATIONAL PRODUCT

Each of the drugs used in this study is approved for human use and has a prior history of use in the treatment of LF.

5.1 Study Drug Background

Albendazole (ALB) has been known to cause degenerative alterations in the tegument and intestinal cells of the worm by binding to the colchicine-sensitive site of tubulin, thus inhibiting its polymerization or assembly into microtubules [9]. The loss of cytoplasmic microtubules leads to impaired uptake of glucose by larval and adult stages of the parasite, and depletes glycogen stores. Degenerative changes in endoplasmic reticulum and mitochondria of the germinal layer, and the subsequent release of lysosomal enzymes result in decreased production of adenosine triphosphate, which is the source of energy required for survival of the helminth. Due to diminished energy production, the parasite is immobilized and eventually dies. Adverse events are uncommon in persons who are treated with a single dose of albendazole (apart from AEs that result from parasite death). Some patients report mild gastrointestinal AEs such as nausea after ingesting the tablet.

Ivermectin (IVM) is an avermectin compound of macrocyclic lactones derived from the bacterium *Streptomyces avermitilis* [10]. The mechanism by which IVM kills LF microfilariae is not known with certainty, but the drug interferes with glutamate gated ion channels that can affect parasite contractility and release of immunomodulatory molecules by the parasite [11]. IVM also has a direct effect on the central nervous system and muscle function of worms as it enhances strength of inhibitory neurotransmission pathways. The main concern with the use of IVM in animals and humans is neurotoxicity, which can be manifest as ataxia. Neurotoxicity has not been observed in humans given single dose IVM for LF or other parasitic infections. IVM has been used to treat millions of people with LF and onchocerciasis. Peak IVM serum concentrations are reached approximately 4-5 hours after administration. The half-life of IVM in various populations ranges from 12 to 56 hours [12]. There is no evidence of drug: drug interaction between ALB and IVM [13]. IVM can cause nausea, dizziness and occasionally pruritus, but these are infrequent, transient and usually mild. Serious adverse events have occurred in patients with heavy *Loa loa* infections.

DEC (diethylcarbamazine citrate) is an anthelminthic drug that is structurally distinct from ALB and IVM [14]. DEC inhibits arachidonic acid metabolism by LF, and inducible nitric oxide synthase and the cyclooxygenase pathway may be essential for activity *in vivo* [14]. DEC also has anti-inflammatory properties. The mechanisms of action of DEC remain poorly understood. Its ability to kill MF and adult worm depends on the host immune responses since the drug has little direct activity on parasites in vitro. The drug has potent activity against LF microfilaria. DEC has about 50-70% [15] efficacy in killing or sterilization of adult worms. The drug is rapidly absorbed from the gastrointestinal tract, has a serum half-life of 12 to 14 hours, and is excreted

in the urine with little modification by liver metabolism. Adverse events from DEC are unusual apart from those that result from killing filarial worms.

5.2 Product Supply and Storage

Only WHO approved drugs will be used in this study. DEC and albendazole will be provided by the Haiti Lymphatic Filariasis Elimination Program through the WHO. A request will be submitted to the manufacturer Merck to provide ivermectin for the present study. Alternatively WHO approved generic ivermectin may be purchased.

All three study drugs are approved and distributed globally by WHO as part of GPELF. Detailed information for each drug is available from the pharmaceutical manufacturer. All products should be maintained at ambient temperature, if possible <30 °C.

5.3 Triple Drug Therapy (IDA) and Two-Drug Therapy (DA)

The triple-drug combination will consist of a single dose of ivermectin (200 µg /kg), DEC (6mg/kg) and albendazole (flat dose of 400 mg). The two-drug combination will consist of a single dose of DEC (6mg/kg) and albendazole (flat dose of 400 mg). Study personnel will directly observe oral administration of drugs. Drugs will be given after the informed consent has been obtained. The study population will be encouraged to eat before swallowing the medicine (without chewing the tablets) with a glass of water. Vomited doses will be replaced. Drug administration will be supervised (directly observed treatment or DOT) to ensure that all enrolled individuals swallow the drugs.

6 SAFETY REPORTING AND SAFETY MONITORING

The post-treatment assessment team will be composed of individuals with basic medical training who are able to perform a medical history and a basic physical examination (physicians, local health workers, nursing and/or medical students). Physicians from the area will be available to assist in the evaluation and management of adverse events.

6.1 Definitions

Adverse Event (AE)

Any untoward medical occurrence in a clinical investigation participant who has received a study product intervention and that does not necessarily have to have a causal relationship with the study product. An AE can, therefore, be any unfavorable and unintended sign (including an abnormal laboratory finding, for example), symptom, or disease temporally associated with the use of a study medicinal product, whether or not considered related to the study medicinal product.

An AE does not include:

- Medical or surgical procedures (e.g. surgery, tooth extraction, transfusion). The condition that leads to the procedure is an adverse event
- Pre-existing diseases or conditions or laboratory abnormalities present or detected prior to the screening visit that do not worsen

Serious Adverse Event (SAE)

A SAE is any adverse event that results in any of the following outcomes:

- Death;
- Life-threatening (immediate risk of death);
- Inpatient hospitalization or prolongation of existing hospitalization;
- Persistent or significant disability or incapacity;
- Congenital anomaly/birth defect;
- Important medical events that may not result in death, be life threatening, or require hospitalization may be considered a serious adverse event when, based upon appropriate medical judgment, they may jeopardize the participant and may require medical or surgical intervention to prevent one of the outcomes listed in this definition. Examples of such medical events include allergic bronchospasm requiring intensive treatment in an emergency room or at home, blood dyscrasia or convulsions that do not result in inpatient hospitalization, or the development of drug dependency or drug abuse.

Unexpected

An adverse reaction, the nature or severity of which is not consistent with the applicable product information (e.g., Package Insert).

Expedited Safety Report

Documentation in appropriate form and format summarizing an SAE that meets expedited safety reporting criteria, submitted within the required reporting time frame of applicable regulatory authorities and/or IRBs/IECs of participating countries.

6.2 Assessment of Adverse Events

Adverse event monitoring will be performed approximately 24 and 48 hours following drug administration (late afternoon and evenings following treatment, house visit #3 and 4, Appendix 1). All dosed participants will be followed for adverse events through Day 7.

Evaluations will be documented on pre-printed Patient Monitoring forms (Appendix 3) using the scoring instructions for AEs (Appendix 4) or entered directly into an electronic form using tablet computers.

Most adverse events after MDA are associated with killing of MF and are seen in the first 12-24h following treatment. However, occasional adverse events related to adult worm death may be delayed by several days.

To capture these adverse events and to assure that any systemic adverse events that occurred earlier have resolved, study personnel will also visit study villages daily on days 3 through 7 after treatment (passive AE monitoring). Individuals with AEs that interfere with activities of daily living (grade 2 or higher) will have more detailed assessments that will include a brief physical examination (including measurement of temperature, blood pressure and pulse).

6.3 Serious Adverse Event (SAE) Assessment and Management

Study participants with definite or suspected serious AEs (any event ≥ grade 3) will be referred to a physician or appropriate health care professional for evaluation. These evaluations will be documented with special adverse event evaluation forms (Appendix 5), following the instructions (Appendix 5a).

An SAE may qualify for reporting to regulatory authorities if the SAE is possibly attributable to one or more of the study drugs, and is unexpected based on the Company Core Safety Information.

The investigator should notify the Institutional Review Board (IRB) or Ethics Committee (EC) as soon as is practical, of serious events in writing where this is required by local regulatory authorities, and in accordance with the local institutional policy.

6.4 Reporting of Pregnancy

Pregnancy is an exclusion criteria for this study. Although not AEs, pregnancies are reportable events. In the event that a pregnant woman is inadvertently dosed efforts will be made to follow-up and ascertain and report the pregnancy outcome (e.g., any premature terminations, elective or therapeutic, and any spontaneous abortions or stillbirths, as well as the health status of the mother and child including date of delivery and infant's gender and weight). Any pregnant woman inadvertently dosed who has a miscarriage or spontaneous abortion within the week of follow-up will be reported as an SAE.

6.5 Safety Monitoring by the Oversight Committee

A DSMB consisting of 4 experts (including 3 physicians) knowledgeable in neglected tropical diseases will be in place to monitor the safety data per country and across countries participating in the DOLF project.

6.6 Country Specific Safety Reporting

The medical monitor will forward SAE reports to the PI in Haiti within 48 hours, and to the data safety review board (DSRB). All SAEs will be reported to the Haitian Bioethical Committee within 7 days.

7 CLINICAL MANAGEMENT OF EVENTS

Individuals who have basic medical training (physicians and/or nursing or medical students) and who are able to complete and pass a training course will be responsible for the initial adverse event evaluations.

In the case of mild symptomatic reactions local health workers/study personnel will provide antipyretics/analgesics and anti-allergic agents at the time of follow-up. It is anticipated that the majority of adverse events will resolve within a day or two and will not require treatment. In the initial adverse event monitoring if any of the following are noted a physician will be notified to evaluate the participant for a potential serious adverse event:

- Participant reports they are unable to participate in their normal daily activities
- Participant has or reports a temperature >39ºC
- Participant has or reports a significant drop in blood pressure
- Participant has other significant objective findings that should be referred to a physician

All grade 3, 4 and 5 events or overnight hospitalization will require completion of the Adverse Event Evaluation and Report Form (Appendix 5). The physician will provide any required immediate treatment and facilitate admission into the hospital or health center as deemed appropriate.

7.1 Adverse Event Monitoring and Management

Adverse Event monitoring and management will follow or exceed WHO guidelines. Participants will be visited on the two days following treatment by study personnel with medical training. Formal assessment of adverse events (with a standard form) will take place on days 1 and 2 and later if symptoms persist or start late.

Study personnel will use the toxicity table (Appendix 4) to score adverse events for severity. Study personnel will visit each study area daily for 7 days following MDA treatment to manage any adverse events as follows:

7.1.1 Mild Localized Symptoms

Participants who develop painful lymphadenopathy, scrotal pain or painful swelling or nodules along lymphatic vessels will be treated with acetaminophen or ibuprofen.

7.1.2 Moderate to Severe Localized Adverse Events

Participants with more severe local adverse effects (≥ Grade 3, Appendix 4) like acute swelling or severe scrotal pain that is not relieved by acetaminophen will be transported by study personnel to the medical facility identified for the study for evaluation by one of the physicians or other qualified medical personnel involved in the study. If necessary, participants will be transferred (after stabilization) to the Departmental Hospital.

7.1.3 Moderate Systematic Adverse Events

Subjects who develop commonly observed symptoms of fever, myalgia, or headache will be advised to rest and will be treated with acetaminophen. Subjects who develop lightheadedness or dizziness will be advised to remain at bed rest until the symptoms have resolved. Subjects who develop nausea or vomiting will be advised to take a clear liquid diet. Antihistamine will also be available for persistent cases.

7.1.4 Moderate to Severe Systemic Adverse Events

Participants with more severe systemic adverse effects (fever over 39 C > 72 hours, other adverse events ≥ Grade 3, syncope, jaundice, or any condition that might require hospitalization) will be transported by study personnel for physician or other qualified medical personnel evaluation at the medical facility identified for the study. If necessary, participants will be transferred (after stabilization) to a local hospital. Serious adverse events will be followed until resolution.

7.2 Rapid Response Teams for Management of Adverse Events

Medical teams will be located at strategic places close to the study sites. Participants, and persons involved in the study (inclusion process and AE monitoring) will be informed about the location and phone numbers of these teams so that they can report directly to these teams if necessary. These teams will be in position from the day of drug administration until the completion of operations.

8 STATISTICAL CONSIDERATIONS

All participants receiving study drug will be included in both the safety and efficacy analysis

8.1 Safety

The sample size of 3000 participants in each arm in Haiti will contribute to the total sample size for the project. The WHO requires a total of 10,000 participants to detect a SAE rate of 0.1% for each of the treatment regimens and recruitment in other countries (e.g., India, Fiji, Indonesia, Papua New Guinea, and Sri Lanka) is planned to contribute to the overall sample size required.

It is well known that systemic AEs are related to killing of MF and that the severity of AEs is related to MF counts. Since MF rates in the study area are relatively low, the Haiti study alone will not be powered to compare rates of SAEs between MDA regimens.

The primary endpoint for safety studies will be the rates of SAEs that occur in infected and in uninfected participants within the first 7 days post MDA. Total AEs will be a secondary endpoint for the study.

8.2 Efficacy

Assuming an MF-prevalence of 1% in the study population at baseline, the survey is expected to detect at least 30 MF positive participants in each arm. A minimum of 21 (70%) of these MF-positive participants in each arm will be retested at 12 months post-treatment for antigenemia and microfilaremia. This sample size is adequate to demonstrate superiority of the IDA regimen (assumptions: 90% reduction in MF prevalence after IDA and 60% reduction after DA, 80% power for detecting an effect size of 30%). The primary endpoint for efficacy will be complete clearance of MF 12 months post MDA. Clearance of filarial antigenemia at 12 months will be a secondary endpoint for the efficacy analysis.

8.3 Enrolling Additional Participants

It is possible that recruitment in other countries may be less than anticipated. In this case the number of participants enrolled in this study may need to be increased to make up for the loss in another country. The number of additional people enrolled will be no more than is necessary to reach the total of 10,000 participants treated with IDA. In this situation the principal investigators will inform the ethics review committees of the expanded enrollment.

9 DATA HANDLING/RECORD KEEPING/SOURCE DOCUMENTS

Data will be collected using a tablet based system, pre-loaded with study templates. Field teams will be trained in the use of the instruments and data will be uploaded as entries are completed.

9.1 Types of Data Collected

Enrollment Data will include (Appendix 2):

- Site Identification
- Participant Identifier
- Informed Consent Date
- Demographic Information
- Pregnancy/last menstrual period
- Medical History
- Presence of hydrocele and lymphedema
- Bed net and window screen use
- History of prior MDA treatment
- Pre-treatment adverse event assessment
- Limited Physical Exam

Laboratory Results

- FTS (filarial antigen test)
- FTS score
- MF slide (including MF count)

Participant Monitoring Forms (24 & 48 hour post treatment):

- Adverse Event Assessment
- Physical Examination, as appropriate

Adverse Event Evaluation and Report (Appendix 5)

- Participant Identification
- MDA Treatment
- Concomitant medication taken at the time of the MDA
- AE Description,
- Start and Stop Date
- Outcome
- SAE Evaluation and causality to MDA (definite, probable, possible, or unrelated)

9.2 Study Records Retention

Study documents will be retained for a minimum of three (3) years after the last participant has completed the study. These documents will be retained for a longer period, however, if required by local regulations. No record will be destroyed without the written consent of DOLF.

Each participating site will maintain appropriate medical and research records for this trial, in compliance with ICH E6, Section 4.9, regulatory and institutional requirements for the protection of confidentiality of participants. Each site participating in this study will permit authorized representatives of the sponsor and regulatory agencies to examine (and when required by applicable law, copy) clinical records for the purposes of clinical site monitoring, quality assurance reviews, audits, and evaluation of the study safety and progress.

9.3 Source Documents

This study will use both paper and electronic source and this may vary by location due to local availability. All sites will be provided with hard copy data collection forms derived from the electronic case report form (CRF). If data is first entered on paper, the study staff will enter the data into the electronic capture system.

10 RESPONSIBILITIES

10.1 Investigator Responsibilities

10.2 Good Clinical Practice

The investigator will ensure that the basic principles of Good Clinical Practice are followed along with the appropriate laws and regulations of the country in which the research is conducted.

10.3 Institutional Review Board (IRB)/Ethics Committee (EC)

The protocol and any accompanying material to be provided to the participants, such as the informed consent, will be submitted to the EC for review and approval. Approval from the ethics committee must be obtained before starting the study and should be documented in correspondence to the investigator.

Any modifications to the protocol after receipt of the IRB or EC approval must be submitted to the committee for approval prior to implementation.

10.4 Informed Consent

It is the responsibility of the investigator to obtain written informed consent from each individual participating in the study after adequate explanation of the aims, methods, objectives and potential risk of any study related procedures. The investigator must use an IRB/EC approved informed consent form (Appendix 6). The investigators will accept either signed (cursive) or printed signatures or a witnessed mark in the case of illiterate study participants on the consent form.

Only the principal investigators or study staff authorized and certified to obtain consent will consent participants for this study. Only individuals who have signed the consent form and meet eligibility criteria will be enrolled in the study.

Entry into the study and participation will be strictly voluntary. It will be made clear that refusal to participate or a decision to withdraw can occur at any time throughout the course of the study and will not influence their rights or the care they receive at local health facilities. Potential participants will be told that all of their health information will be confidential and that records will be coded without personal identifiers before they are shared with statisticians or project scientists outside of the village/region/country. They will also be told that no monetary or other gains are offered in exchange for participation apart from compensation for time and reimbursement of travel expenses as described above.

10.4.1 Informed Consent Training

The PI, co-PIs and the site project coordinator will conduct on-site training sessions for Haitian study personnel who will be collecting study information, specimens, and obtaining consent from participants in the study. Each step of the study will be explained in detail to the local study personnel. The basic principles of informed consent process, documentation of informed consent, protection of participants' rights, confidentiality, and handling of data will be covered in these training sessions. Study personnel will be monitored by the on-site project coordinator on a regular basis to ensure compliance with the principles of informed consent. The investigators and study personnel who will obtain consent from study participants will also receive training in the informed consent process and good clinical practices (GCP).

10.4.2 Country Specific ICF Information

The study will be discussed with Ministry of Public Health representatives and senior community members to assess both feasibility and community acceptability of the study design and field procedures. Community leaders will first give their authorization to include their community in the study.

All consent forms and study information material will be translated in Creole. All study personnel involved in the inclusion process of participants and in AE monitoring will be fluent in Creole and French. The informed consent process recognizes the community and cultural values of Haiti and the commune of Quartier Morin where the study participants reside. Extensive discussion of risks and possible benefits of participation in this study will be provided to the study participants.

Formal, written informed consent will be obtained for individuals willing to participate in the study. The Haitian Ethical Committee (Comite National de Bioéthique) will approve the informed consent form. The participant will be asked to read, or have read to them, and review the informed consent documents. Upon reviewing the document, the investigator and/or study staff will explain the research study to the participant and answer any questions that may arise. The investigators will accept either a participant signed (cursive) or printed signatures or a witnessed signed (cursive) or printed signatures if the study participant is illiterate.

Only the principal investigators or study staff authorized to obtain consent will consent participants for this study. Only individuals who have signed the consent form and meet eligibility criteria will be enrolled in the study.

10.5 Participant Privacy

Privacy of the study participants will be maintained by assigning study participants a unique study identification number (UNID). All data, blood samples and laboratory results will be recorded and analyzed by UNID with no personal identifiers. All information collected, including demographic information about enrolled participants will be kept confidential and available only to the investigators and authorized study personnel such as the data manager.

Though most data will be collected on tablets, all written forms (i.e., consent and any paper data collection forms) will be stored in a designated locked area with limited control. All forms will be labelled and filed in cabinets with the study protocol number, PI's names and collection dates. These cabinets will be metal and have functioning locks. Keys will be kept with the Project Coordinator. All electronic devices on which data are entered will be password protected. PIs and/or the Project Coordinator will authorize access. The paper forms will be stored for the duration of the study plus three years per IRB protocol for primary data storage.

10.6 Data Ownership

The data are the property of MSPP. The Principal Investigators, Co-investigators and key personnel may use the results of this study for publications, presentations at scientific meetings or as preliminary data for subsequent grant applications. Confidentiality of study participants will be maintained by not using names or personal identifiers. MSPP will provide de-identified data from the study to DOLF for use in publications and presentations that present results across different study sites. At least one Haitian researcher will be included as an author for any publications with data from Haiti.

The study site Project Coordinator will permit access to all documents and records that may require inspection by the funding agencies, governmental regulatory agencies, institutional review boards or its authorized representatives.

11 PUBLICATION POLICY

Manuscripts should be submitted for publication no later than one year following the date of the “last patient/last visit”. This study includes follow-up data collection past the primary end point, including acceptability and efficacy results. It is not necessary to wait for the follow-up studies to be completed in order to publish the primary safety data.

Endemic country investigators have an obligation to publish the results of DOLF studies conducted in their country. These results benefit the national NTD programs and the citizens of the country where the study was completed. DOLF collaborating institutions are willing to help their endemic country partners with the data analysis, manuscript preparation, publication fees, etc. However, the lead author should be an investigator from the country where the study was performed.

DOLF scientists will be responsible for publishing the results from the aggregated data that combines the results from multiple study sites. The purpose of these manuscripts is to consider the similarities and differences in results obtained in different countries. These publications will not include as much detailed data or analyses as the country specific publications. Publications that report multi-country results will have at least one co-author from each country included in the manuscript.

12 LITERATURE REFERENCES

1. *World Health Organization (1997). Elimination of lymphatic filariasis as a public health problem - resolution of the executive board of the WHO.*
2. Thomsen, E.K., et al., *Efficacy, Safety, and Pharmacokinetics of Coadministered Diethylcarbamazine, Albendazole, and Ivermectin for Treatment of Bancroftian Filariasis.* Clin Infect Dis, 2015.
3. Gyapong, J.O., et al., *Treatment strategies underpinning the global programme to eliminate lymphatic filariasis.* Expert Opin Pharmacother, 2005. **6**(2): p. 179-200.
4. *Global programme to eliminate lymphatic filariasis: progress report, 2014.* Wkly Epidemiol Rec, 2015. **90**(38): p. 489-504.
5. Oscar, R., et al., *Haiti National Program for the elimination of lymphatic filariasis--a model of success in the face of adversity.* PLoS Negl Trop Dis, 2014. **8**(7): p. e2915.
6. *World Health Organization (2002). Global Programme to Eliminate Lymphatic Filariasis. Annual Report on Lymphatic Filariasis. Geneva: WHO*. Available from: http://www.filariasis.org/pdfs/Press%20Centre/Reports/LF_Annual_Report_2002.pdf.
7. de Rochars, M.B., et al., *The Leogane, Haiti demonstration project: decreased microfilaremia and program costs after three years of mass drug administration.* Am J Trop Med Hyg, 2005. **73**(5): p. 888-94.
8. *Envision (2015). Haiti Work Plan, FY 2016, Project Year 5, October 2015-September 2016. Available from: http://ntdenvision.org/resource/envision_reports_to_usaid/haiti_fy16_workplan.*
9. Horton, J., *Albendazole: a review of anthelmintic efficacy and safety in humans.* Parasitology, 2000. **121 Suppl**: p. S113-32.
10. Goa, K.L., D. McTavish, and S.P. Clissold, *Ivermectin. A review of its antifilarial activity, pharmacokinetic properties and clinical efficacy in onchocerciasis.* Drugs, 1991. **42**(4): p. 640-58.
11. Edwards, G., *Ivermectin: does P-glycoprotein play a role in neurotoxicity?* Filaria J, 2003. **2 Suppl 1**: p. S8.
12. Ottesen, E.A. and W.C. Campbell, *Ivermectin in human medicine.* J Antimicrob Chemother, 1994. **34**(2): p. 195-203.
13. Awadzi, K., et al., *The co-administration of ivermectin and albendazole--safety, pharmacokinetics and efficacy against Onchocerca volvulus.* Ann Trop Med Parasitol, 2003. **97**(2): p. 165-78.
14. Ottesen, E.A., *Efficacy of diethylcarbamazine in eradicating infection with lymphatic-dwelling filariae in humans.* Rev Infect Dis, 1985. **7**(3): p. 341-56.
15. Noroes, J., et al., *Assessment of the efficacy of diethylcarbamazine on adult Wuchereria bancrofti in vivo.* Trans R Soc Trop Med Hyg, 1997. **91**(1): p. 78-81.

13 LIST OF APPENDICES

Appendix 1: Study Flow Diagram (Country Specific)

Appendix 2: Participant Enrollment Form [Example]

Appendix 3: Participant Monitoring Form [Example]

Appendix 4: Guide to Assigning Adverse Event Severity

Appendix 5: Adverse Event Evaluation and Report Form (AEERF) [Example]

Appendix 5a: Required Reporting & Guidelines for SAE(s)

Appendix 6: Information Sheet and Informed Consent Form [Example]

APPENDIX 1: STUDY FLOW DIAGRAM (COUNTRY SPECIFIC)

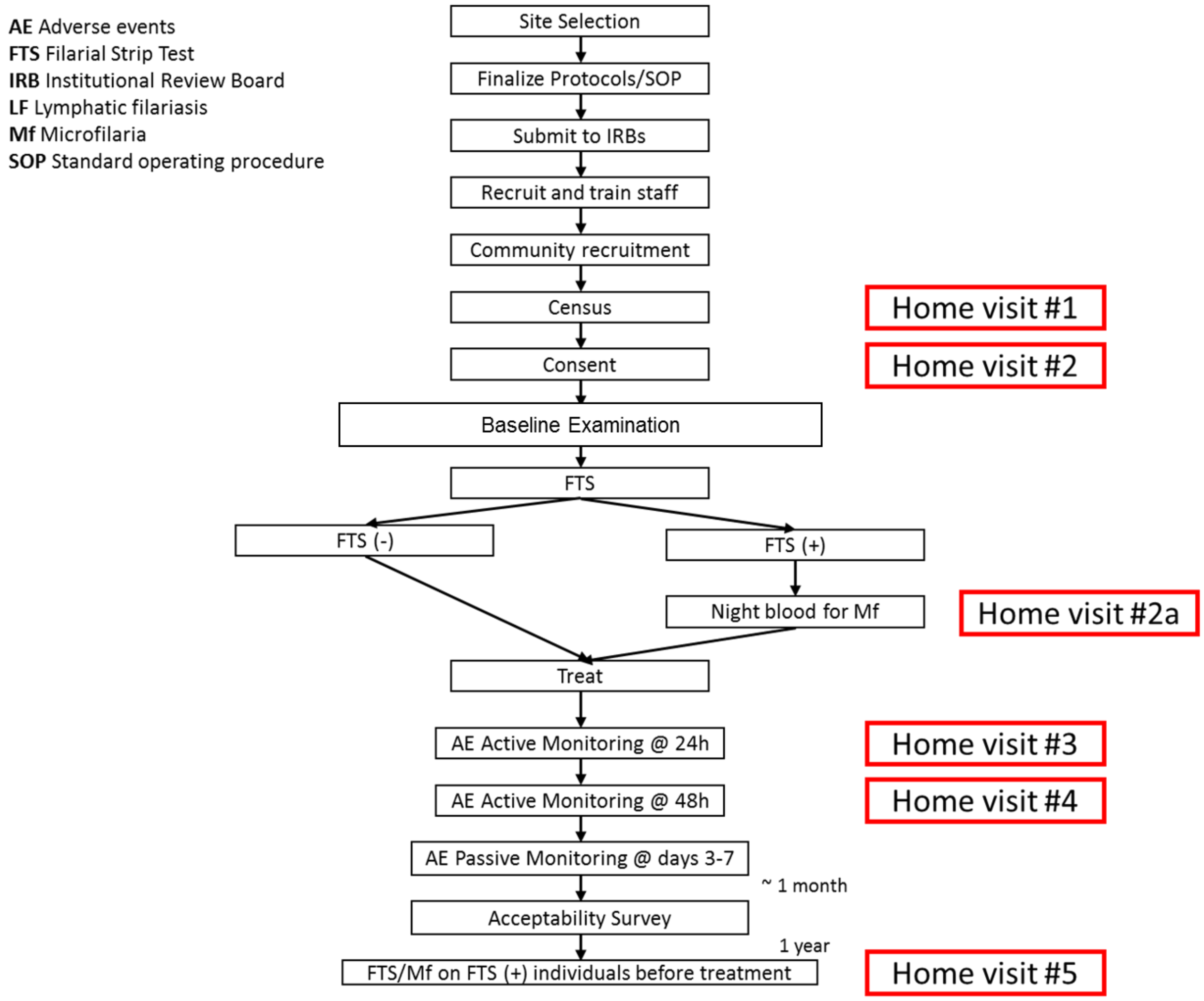

APPENDIX 2: PARTICIPANT ENROLLMENT FORM [EXAMPLE]

Participant ID (Barcode):

1. SITE INFORMATION

Team (**required**):	Data Entry Clerk ID (**required**):
Enrollment Date (**required**) (DD-MM-YYYY):	
Consent Method (**required**)<br>☐Self<br>☐Parent<br>☐Other guardian (specify):<br>☐Teacher<br>☐No consent (STOP)	Enrollment Location<br>☐Home<br>☐School<br>☐Village meeting point
Enrollment Village:	

2. PARTICIPANT INFORMATION AND MEDICAL HISTORY

Gender: ☐M ☐F	*Note: if exact date is not known, birth year is sufficient*			Age (Years):
	Birth Day (DD):	Birth Month (MM):	Birth Year (YYYY):	
Village of Residence:		House Number:		
Participant ID (Barcode):<br>(affix barcode at the top of each form AND write in ID number at top of each page)				
Females only: **When was the date of your last menstrual period? (*read options)***<br>☐Definitely less than 4 weeks ago<br>☐Post-menopause<br>☐4 weeks of longer (STOP)<br>☐Uncertain (STOP)				
Males only: **Do you have swelling or enlargement of your scrotum?** ☐Yes ☐No				
Males only: **Do you feel pain in your testicles or scrotum?** ☐Yes ☐No				
**Do you have any of the following chronic medical conditions? (*read options*)**<br>☐Hypertension (high blood pressure)<br>☐Asthma or chronic lung disease<br>☐Chronic kidney disease (renal insufficiency)<br>☐Diabetes<br>☐Other (specify):				
**Do you have swelling in your arms or legs (lymphedema)?** ☐Yes ☐No				
If participant reports lymphedema exam them and confirm presence of edema.<br>Left arm ☐No edema ☐Yes edema				

APPENDIX 2: PARTICIPANT ENROLLMENT FORM [EXAMPLE]

Participant ID (Barcode):

Left leg	☐No edema	☐Yes edema
Right arm	☐No edema	☐Yes edema
Right leg	☐No edema	☐Yes edema

**Did you use a bed net last night?**	☐Yes ☐No
**Does your house have screens on the windows?**	☐Yes ☐No
**Do you spray indoors to prevent mosquitos?**	☐Yes ☐No

Did you swallow medicines during MDA treatment for filariasis in the last twelve months? (If YES enter the date)

☐Yes Date of last MDA (MM-YYYY):___________ ☐Do not remember date
☐No
☐Uncertain
☐N/A—no MDA distributed

Have you ever taken the following medication called…?

**Albendazole?**	**Ivermectin?**	**DEC?**
☐Yes ☐No ☐Don't know	☐Yes ☐No ☐Don't know	☐Yes ☐No ☐Don't know
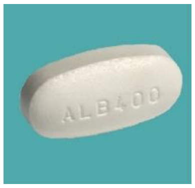	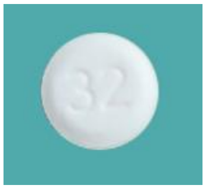	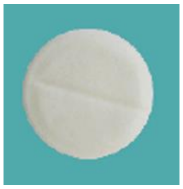

3. AE ASSESSMENT

Today, were you too sick to work or go to school?
☐Yes (**exclude, but finish the pre-treatment assessment**)
☐No

EXPLAIN: ***Now I will ask you some questions about your health during the past two days.***

In the past 2 days have you experienced…?

..Fever	☐Yes ☐No
..Dizziness, giddiness, or fainting	☐Yes ☐No
..Confusion	☐Yes ☐No
..Drowsiness	☐Yes ☐No
..Headache	☐Yes ☐No
..Cough	☐Yes ☐No
..Difficulty breathing (wheezing or dyspnea)	☐Yes ☐No
..Nausea	☐Yes ☐No
..Vomiting	☐Yes ☐No
..Diarrhea	☐Yes ☐No

APPENDIX 2: PARTICIPANT ENROLLMENT FORM [EXAMPLE]

Participant ID (Barcode):

..Stomach pain ☐Yes ☐No<br>..Unusual swelling, beyond baseline lymphedema ☐Yes ☐No<br>Specify Location: ☐Arm ☐Leg ☐Breast ☐Scrotum<br>..Joint or muscle pain ☐Yes ☐No<br>..Weakness ☐Yes ☐No<br>..Swelling or pain in your armpit or groin ☐Yes ☐No<br>..Men only: pain in you testicles or scrotum ☐Yes ☐No<br>..Itchy skin ☐Yes ☐No<br>..Rash (specify location):__________ ☐Yes ☐No<br>Is the rash in the spaces between your fingers? ☐Yes ☐No<br>..Other illness or symptoms (specify):__________ ☐Yes ☐No
**Have you ever suffered from scabies?** (*note: use local name for scabies*)<br>☐Yes<br>☐No<br>☐Don't know
Additional notes or comments:

4. EXAMINATION

Team (**required**):	Clinician (**required**):
Data Entry Clerk ID (**required**):	

**Measurements**	**Values / status**
Height (cm)	
Weight (kg)	
BMI (calculated)	
Scabies	☐Yes ☐No
If Yes, please take photograph	

APPENDIX 3: PARTICIPANT MONITORING FORM [EXAMPLE]

FOR: Day 1 & 2, if needed days 3-7

Participant ID (Barcode):

Use this form for active monitoring of adverse events on day 1 (24 hours) and 2 (48 hours) following therapy, as well as for recording symptoms reported by those presenting with complaints on days 3-7 post-treatment.

Team (**required**):	Clinician (**required**):
Data Entry Clerk ID (**required**):	

1. PARTICIPANT INFORMATION

Gender: ☐M ☐F	Age (Years):	Village of Residence:
Treatment Village:		Treatment Date (DD-MM-YYYY):

2. ASSESSMENT INFORMATION

*Day 1 and 2: **All** participants should be asked **all** the questions in Table 1.*
*Days 3-7: Any participant who presents with a complaint should be asked **all** the questions in Table 1*

Table 1: Reported Symptoms

- *Record a symptom grade from 0-5 for each day on which the participant experienced symptoms.*
- *For participants reporting ANY symptom, complete every questions in Table 1.*
- *Refer to the Appendix 4 for symptom-specific scoring criteria.*
- *Anyone with a symptom typed in **bold** needs to have Table 2 completed.*

Symptom Grading
0 = No adverse event or within normal limits
1 = Mild adverse event, does not interfere with work or school
2 = Moderate adverse event, interferes with work or school at least 1 day
3 = Severe and undesirable adverse event; interferes with ADL, requires medical assessment
4 = Potentially life-threatening or disabling adverse event; requires transfer to medical facility
5 = Death

Symptoms/Signs	**Post-treatment day(s) on which symptoms or signs were present**						
**Since you took the tablets have you experienced…?**	**Day 1**	**Day 2**	**Day 3**	**Day 4**	**Day 5**	**Day 6**	**Day 7**
..**Fever**							
..**Dizziness, giddiness, or fainting**							
..**Confusion**							
..**Drowsiness**							

APPENDIX 3: PARTICIPANT MONITORING FORM [EXAMPLE]

Participant ID (Barcode):

..Headache							
..Cough							
..**Difficulty breathing (wheezing or dyspnea)**							
..Nausea							
..Vomiting							
..Diarrhea							
..Stomach pain							
..Unusual swelling, beyond baseline lymphedema (specify location below)							
Arm							
Leg							
Breast							
Scrotum							
..Joint or muscle pain							
..Weakness							
..**Swelling or pain in your armpit or groin**							
..Men only: pain in your testicles or scrotum							
..Itching skin							
..**Rash (specify location and brief description):**							
..Other illness or symptoms (specify):							

If there is any symptom grade ≥ 2, you must notify the supervising medical officer and the participant must be evaluated by the medical team.

APPENDIX 3: PARTICIPANT MONITORING FORM [EXAMPLE]

Participant ID (Barcode):

**Table 2: Physical Examination**							
• *You must complete this table for any participant reporting any **bolded** symptom in Table 1 OR for any symptom **grade ≥2*** • *Record the result under the column that corresponds to the day the assessment was taken.*							
	**Post-treatment day(s)**						
**Measurements**	**Day 1**	**Day 2**	**Day 3**	**Day 4**	**Day 5**	**Day 6**	**Day 7**
Height (cm)							
Weight (kg)							
BMI (calculated)							
Temperature							
Blood pressure, sitting							
Blood pressure, lying down (*measure only if sitting systolic BP <100)*							
**Post-Exam Adverse Event Grade** (Assign grade of 0-5 for the adverse reactions below based on physical exam. See Appendix 4 under "post-exam assessment" for specific grading criteria)							
Allergic reaction							
Hypotension (low blood pressure)							
Lymphangitis (streaks of redness, warmth, and swelling in arms or legs)							

APPENDIX 4: GUIDE TO ASSIGNING ADVERSE EVENT SEVERITY

(*Grade 0 = no symptoms; grade 5 = death from adverse event*)

**Symptoms/Signs**	**Grades**			
	**1. Mild**	**2. Moderate**	**3. Severe**	**4. Life-threatening**
Fever (non-axillary temperatures only)	38.0 – 39.0ºC	39.1 – 40.0ºC	> 40.0ºC	> 40.0ºC for > 48 hrs
Dizziness, giddiness, or fainting	Mild, not interfering with work or school	Moderate, unable to work or attend school for 1 day, but no fainting	Any loss of consciousness (fainting)	-
Confusion or excess drowsiness*	Mild, not interfering with work or school	Moderate; confusion or drowsiness interfering with ability to work	Confusion, loss of memory, or sleepiness interfering with activities of daily living	Delerium, inability rouse, or coma
Fatigue	Mild, not interfering with work or school	Moderate, unable to work or attend school at least 1 day	Unable to perform activities of daily living, > 1day	Required hospitalization
Headache	Mild pain not interfering with work or school	Moderate pain; pain or analgesics interfering with ability to work or attend school	Severe pain; pain or analgesics interfering with activities of daily living	Disabling, duration > 48 hr
Cough	Mild, relieved by non-prescription medication	Requiring narcotic antitussive	Severe cough or coughing spasms, poorly controlled by treatment	Hospitalization or respiratory failure requiring mechanical ventilation
Difficulty breathing (wheezing or dyspnea)	Mild, not interfering with work or school	Moderate, unable to work or attend school for 1 day	Severe, more than 1 day and required transfer to clinic or hospital	Hospitalization or respiratory failure requiring mechanical ventilation
Nausea	Able to eat	Oral intake significantly decreased	No significant intake, requiring IV fluids	-
Vomiting	1 episode in 24 hours over pretreatment	2-5 episodes in 24 hours over pretreatment	≥ 6 episodes in 24hours, or need for IV fluids (0upatient)	Hemodynamic collapse or overnight hospitalization

Appendix 4: GUIDE TO ASSIGNING ADVERSE EVENT SEVERITY

(G*rade 0 = no symptoms; grade 5 = death from adverse event*)

Symptoms/Signs	Grades			
	**1. Mild**	**2. Moderate**	**3. Severe**	**4. Life-threatening**
Diarrhea	Increase of < 4 stools/day over pre-treatment	Increase of 4-6 stools/ day, or nocturnal stools	Increase of ≥ 7 stools/ day or need for outpatient parenteral support for dehydration	Physiologic consequences wth hemodynamic collapse or requiring hospitalization
Abdominal pain	Mild pain not interfering with work or school	Moderate pain; pain or analgesics interfering with ability to work or attend school	Severe pain; pain or analgesics interfering with activities of daily living	Disabling, duration > 48 hr
Unusual swelling (beyond baseline lymphedema)	Mild, not interfering with work or school	Moderate, unable to work or attend school 1 day	Severe, unable to work/school >1 day	Severe, limiting activities of daily living (unable to walk) > 2 days
Joint or muscle pain	Mild pain not interfering with work or school	Moderate pain; pain or analgesics interfering with ability to work or attend school	Severe pain; pain or analgesics interfering with activities of daily living	Disabling, duration > 48 hr
Swelling or pain in your armpit or groin*	Mild, not interfering with work or school	Moderate, unable to work or attend school 1 day	Severe, unable to work/school >1 day	Severe, limiting activities of daily living (unable to walk) > 2 days
Men only: testicular or scrotal pain	Mild, not interfering with work or school	Moderate, unable to work or attend school 1 day	Severe, unable to work/school >1 day	Severe, limiting activities of daily living (unable to walk) > 2 days
Itching skin	Mild, not interfering with work or school	Moderate, unable to work or attend school 1 day	Severe, unable to work/school >1 day	
Rash	Localized rash (covers only one part of the body)	Diffuse rash (covers multiple parts of the body)	Diffuse rash (covers multiple parts of the body) AND has any blisters or ulcers or mouth sores	Extensive areas with blisters or ulcers OR peeling or blackening of skin
Other illness or symptoms	Mild, not interfering with work or school	Moderate, unable to work or attend school at least 1 day	Unable to perform activities of daily living, > 1day	Required hospitalization

Post-Exam Assessment	Grades			
	**1. Mild**	**2. Moderate**	**3. Severe**	**4. Life-threatening**

Appendix 4: GUIDE TO ASSIGNING ADVERSE EVENT SEVERITY

(G*rade 0 = no symptoms; grade 5 = death from adverse event*)

Acute allergic reaction	Transient rash, drug Fever <38ºC (<100.4ºF)	Urticaria, drug fever ≥38ºC (≥100.4ºF) and/or asymptomatic bronchospasm	Symptomatic bronchospasm, requiring parenteral medication(s) with or without urticaria	Anaphylaxis with hypotension required hospitalization
Hypotension (low blood pressure)	Changes, but not requiring therapy (including transient orthostatic hypotension)	Requiring brief fluid replacement (such as oral rehydration) but not hospitalization	Requiring i.v. fluids without overnight hospitalization. No sequelae.	Required overnight hospitalization for i.v. fluids, or Shock (acidemia and impaired vital organ function due to tissue hypoperfusion)
Lymphangitis	Mild, not interfering with work or school	Moderate, unable to work or attend school 1 day	Severe, unable to work/school >1 day	Severe, limiting activities of daily living (unable to walk) > 2 days

Note on general aspects of grading

0 = No adverse event or within normal limits
1 = Mild adverse event, does not interfere with work or school
2 = Moderate adverse event, interferes with work or school at least 1 day
3 = Severe and undesirable adverse event; interferes with ADL, requires medical assessment
4 = Potentially life-threatening or disabling adverse event; requires transfer to medical facility
5 = Death

Note: Any event ≥ grade 2 requires a medical evaluation and notification of the medical officer. Any grade 3, 4 or 5 event or overnight hospitalization requires an Adverse Event Evaluation and Report Form.

APPENDIX 5: ADVERSE EVENT EVALUATION AND REPORT FORM (AEERF) [EXAMPLE]

Participant ID (Barcode):

Instructions: Complete this form AFTER completing the Participant Monitoring Form for anyone with symptoms or signs of <u>grade 3 or higher</u> *(unable to perform activities of daily living without assistance for at least one day). The purpose of this form is to provide additional information on more severe adverse events and to assist the medical officer in determining whether a Serious Adverse Event (SAE) has occurred. Please refer to Appendix 5a for definitions.*

Clinician (**required**):

1. PARTICIPANT INFORMATION

**Participant ID (Barcode):**			
Gender: ☐M ☐F	Age:_____Years	Weight:_____Kg	Height:_____cm
Village of Residence:			

2. MDA TREATMENT

Treatment Date (DD-MM-YYY)___________<br>Treatment Village:______________________<br>Anything irregular about treatment?<br>☐No ☐Yes (specify):	Medications received<br>☐ Albendazole (dose:____mg)<br>☐ DEC (dose:____mg)<br>☐ Ivermectin (dose:____mg)
**Was this the first time you have ever been treated with one of the MDA medications? If No, explain when and circumstances of prior treatment.**<br>Albendazole ☐Yes ☐No (explain):<br>DEC ☐Yes ☐No (explain):<br>Ivermectin ☐Yes ☐No (explain):	

3. OTHER MEDICATIONS AT TIME OF MDA

Please include prescription and non-prescription medications/supplements/herbal remedies taken within 5 days of the MDA. DO NOT include medications used to treat the SAE.

Medication	Indication	Dose and Frequency	Days on which each medication was taken, relative to MDA (if taken the day of MDA, mark “0”; the day before, mark “-1”; the day after, “+1”, and so forth.)
			-5 -4 -3 -2 -1 0 +1 +2 +3 +4 +5 uncertain
			-5 -4 -3 -2 -1 0 +1 +2 +3 +4 +5 uncertain
			-5 -4 -3 -2 -1 0 +1 +2 +3 +4 +5 uncertain

Appendix 5: ADVERSE EVENT EVALUATION AND REPORT FORM (AEERF) (EXAMPLE)

Participant ID (Barcode):

Medication	Indication	Dose and Frequency	Days on which each medication was taken, relative to MDA (if taken the day of MDA, mark "0"; the day before, mark "-1"; the day after, "+1", and so forth.)
			-5 -4 -3 -2 -1 0 +1 +2 +3 +4 +5 uncertain

4. DESCRIPTION OF THE ADVERSE EVENT

Date of onset (DD-MM-YYYY):	How long after drugs were taken did the event begin?<br>_____hours OR _____days
Clinical signs and symptoms (please describe)<br><br>Do you (the clinician) think this adverse event is/was life-threatening? ☐Yes ☐No	
Was the participant hospitalized? ☐Yes ☐No<br>If yes, indicate<br>1. Date of admission (DD-MM-YYYY):<br>2. Reason for admission:<br>3. Date of discharge (DD-MM-YYYY):<br>4. Clinical course, including drug treatments given to treat adverse event:<br><br>*Attach a copy of any medical records relating to the diagnosis and treatment of the adverse event*	
Laboratory results and diagnostic tests (indicate date, test name, and results):	

Appendix 5: ADVERSE EVENT EVALUATION AND REPORT FORM (AEERF) (EXAMPLE)

Participant ID (Barcode):

5. ADVERSE EVENT OUTCOME (Check only ONE)

☐Recovering/resolving		
☐Not recovered/not resolved		
☐Recovered/resolved	Date: (DD-MM-YYY)	
☐Recovered/resolved with sequelae	Date:(DD-MM-YYY)	Sequelae:
☐Unknown		
☐Fatal (death)	Date:(DD-MM-YYY)<br>Autopsy: ☐Not done ☐Done (*provide report*) ☐Planned ☐ Status Unknown<br>Death certificate: ☐Provided ☐Requested ☐Not available ☐ Status Unknown	

Appendix 5: ADVERSE EVENT EVALUATION AND REPORT FORM (AEERF) (EXAMPLE)

Participant ID (Barcode):

6. CONCLUSIONS (to be completed by the health-care provider)

Presumptive diagnosis:
Do you think this adverse event was caused by the MDA medications? *Refer to Appendix 5a for detailed explanation of choices.*<br>☐ Definitely<br>☐ Probably (explain):<br>☐ Possibly (explain):<br>☐ Unrelated<br>If “unrelated”, what do you believe was the cause of the adverse event?
Does this event meet the criteria for a Serious Adverse Event (SAE*)? Refer to Appendix 5a for detailed definitions of criteria.*<br>☐ Yes, based on the following criteria<br>☐ Death<br>☐ Life-threatening<br>☐ Hospitalization<br>☐ Disability or permanent damage<br>☐ Other serious important medical event: specify<br>☐ No

REPORTER INFORMATION AND SIGNATURES

Investigator Name:	Investigator Signature:	Date:
Reporter Name:	Reporter Signature:	Date:
Reporter’s phone number:	Reporter’s email address:	

APPENDIX 5A: REQUIRED REPORTING GUIDELINE FOR SERIOUS ADVERSE EVENTS

Participant ID (Barcode):

An Adverse Event Evaluation and Report Form (AEERF) should be completed for every severe adverse event (those scoring grade 3 or higher, see Appendix 4). However, a grade 3 or severe adverse event is NOT the same as a Serious Adverse Events (SAE) and the majority of grade 3 adverse events will not be classified as SAE. The term "severe" is often used to describe the intensity (severity) of a specific event (as in mild, moderate, or severe myocardial infarction); the event itself, however, may be of relatively minor medical significance (such as severe headache). This is not the same as "serious," which is based on patient/event outcome or action criteria usually associated with events that pose a threat to a patient's life or functioning. Seriousness (not severity) serves as a guide for defining regulatory reporting obligations.

The AEERF should guide the medical monitor or health care provider evaluating the patient experiencing a severe AE to determine whether a SAE has occurred. All SAE must be reported promptly. (See Safety Reporting Plan for SAE Reporting Timeline)

Required Reporting

A written report or CRF must be sent from the local physician and local medical monitor by email (scanned records) in the stated timeframes to the Country PI, Global Medical Monitor including the Project PI for the events listed below.

Guidelines for Reporting - Standard Reporting Information

The following information should be included in the initial report/CRF (additional information may be requested):

APPENDIX 5A: REQUIRED REPORTING GUIDELINE FOR SERIOUS ADVERSE EVENTS

Minimum Criteria for Reporting

Information for final description and evaluation of a case report may not be available within the required time frames for reporting outlined below. Initial reports should be submitted within the prescribed time as long as the following minimum criteria are met: an ***identifiable patient***; ***an identifiable reporting source***; *and* ***an event or outcome that can be identified as serious***. Follow-up information should be actively sought and submitted as it becomes available.

Complete the following information if available on the initial report and complete a follow-up report as new or additional information becomes available as noted below:

- Description of the event
  Date, time of onset
  Clinical history
  Associated signs and symptoms
  Temporal association with study agent
  Medical management, including rationale
  Pertinent laboratory tests
  Severity – *see definitions or toxicity score*
  Causal relationship to the study drug/vaccine

- Other information
  Relevant past medical history
  Concomitant medications
  Autopsy report or expectation of an autopsy in the case of death

- Outcome of event
  Date, time of resolution, if resolved

- Plans for study participant
  Follow-up
  Treatment of event
  Return to treatment/Contraindicate

- Location/Study Centre

- Reporting Physician

- Verification of notification to IRB and Safety Monitor or DSMB

Definitions

- Adverse Event [Experience] (AE):

Any untoward medical occurrence, including dosing errors, that may arise during administration of study agent, and which may or may not have a causal relationship with the study agent.

- Unexpected Adverse Event [Experience]:

  Any adverse experience that has not been previously observed (i.e., included in the labelling), whether or not the event is anticipated because of the pharmacologic properties of the study agent.

- Serious Adverse Event (SAE):

  Any adverse event occurring at any dose that results in any of the following outcomes:

a. Death

b. Life threatening – defined as an experience that places the patient or participant, in the view of the Investigator, at *immediate risk* of death from the reaction as it occurred. (Note; this does not include a reaction that, had it occurred in a more severe form, might have caused death.)

c. Requires inpatient hospitalization or prolongation of existing hospitalization

d. Results in a congenital anomaly or birth defect

e. Results in a persistent or significant disability or incapacity

f. Important medical events that may not result in death, be life-threatening, or require hospitalization may be considered a serious adverse experience when, based upon appropriate medical judgment, they may jeopardize the patient or participant and may require medical or surgical intervention to prevent one of the outcomes listed in this definition. *(The event might be defined as serious based on progression of grade if Toxicity Tables are being used.)*

Severity

Adverse experience/events should be assessed by the on-site investigator as to their severity and/or intensity.

a. Life threatening

b. Severe: incapacitating with inability to work or do usual activity

c. Moderate: enough discomfort to cause interference with usual activity

d. Mild: awareness of sign or symptom, but easily tolerated

Relationship or Association with Use of Study Agent or Participation in the Study

Causal relationship with the investigational study treatment must be assessed by the on-site investigator using the following or similar terms:

- **Definite** – clear-cut temporal association, with a positive re-challenge test or laboratory confirmation.
- **Probable –** clear-cut temporal association, with improvement upon drug withdrawal, and not reasonably explained by the participant's known clinical state.
- **Possible –** less clear temporal association; other aetiologies are possible.
- **None –** no temporal association with the study drug; related to other aetiologies such as concomitant medications or conditions, or participant's known clinical state.

Other Reporting

Investigators are reminded that they may have other reporting obligations:

- For all studies, there must be compliance with the clinical site Ethics/IRB's policy for reporting adverse events. (As soon as possible for SAEs and as required for AEs.)

APPENDIX 6: INFORMATION SHEET AND INFORMED CONSENT FORM (VERSION 1.2, 22 AUGUST 20116)

Participant ID (Barcode):

Project Title: Community Based Safety Study of 2-drug (Diethylcarbamazine and Albendazole) versus 3-drug (Ivermectin, Diethylcarbamazine and Albendazole) Therapy for Lymphatic Filariasis in Haiti

Part A - Information Sheet (Adult)

Lymphatic filariasis (LF) is a disease carried by mosquitoes and caused by tiny worms in the blood. In Haiti we sometimes call it maladi filarioz. It can cause complications like swelling of the legs (lymphedema) or the scrotum (hydrocele). The Haitian Ministry of Public Health and Population (MSPP) has given treatment for these worms every year in order to stop LF in Haiti. Though treatment has been given annually since 2001, too many people are still infected with LF in your community.

In this consent form the term “you” may refer to you or your child. You are being asked to take part in this study because you live in an area where you may become sick with lymphatic filariasis. You are being asked to consent for yourself and/or your child or children

Why is this Study being done?

This study is being conducted by the Ministry of Public Health and Population (MSPP). We are currently investigating a new combination of three drugs to treat LF. In studies in other countries, this new combination has been shown to be more effective in killing the LF worms more rapidly than the usual combination of two drugs you take for LF annually. It could accelerate the elimination of LF in Haiti and in your community. It consists of the usual drugs you take for LF (diethylcarbamazine and albendazole) plus another drug called ivermectin. This study is done to learn about the side effects people may have when they take these different drugs together to get rid of the LF worm.

As part of this study you will either take the standard combination of two drugs including diethylcarbamazine (DEC) and albendazole (ALB) or the combination of three drugs including diethylcarbamazine (DEC), albendazole (ALB) and ivermectin (IVM). You will be asked to swallow pills; the number of pills will be calculated based on your body weight. When you take these drugs your body may react to the dying worms by developing a fever. You may also feel tired or have body aches for one or two days. This means that the drugs are killing worms. We want to collect information about how your body reacts to these medications, and if taking the three drugs together causes your body to react more strongly compared to when you take the

two drugs. Finally, we want to know if taking three drugs instead of two will completely eliminate the LF worms from your body if you are infected.

How many people will take part in the study?

Approximatively 6,000 men, women and children will take part in this study in Haiti and about 10,000 total in all countries involved

What is involved in the study?

Half of the people who will accept to participate in the study will get 2 drugs (DEC + ALB) and the other half will get 3 drugs (DEC + ALB+ IVM). You will be assigned to either treatment group. Before you receive any medication we will ask you few questions about your health. We might also take your blood pressure and temperature. We will also take few drops of blood by pricking your finger to see if you are infected with LF using a rapid test. If the rapid test is positive we will ask you if we can visit you at night (between 10 pm and 12 am) to get another drop of blood to measure how many worms are in your blood. Even if the rapid test is negative you will be treated with drugs because the test can be negative if the infection is very light.

Some members of the study team will follow up with you 1 and 2 days after you took the drugs. During these visits the study team will ask how you are feeling and might take your temperature and blood pressures. The study team will be in your village 3 to 7 days after you took the drug to make sure you are still feeling well. If you are not well, then you will be examined. If your illness is severe then the study team will have you see a doctor who can provide treatment. The study team will record information about how you are feeling at each visit.

If you tested positive for LF at the beginning of the study, the study team will again collect a small amount of blood from your finger 12 months after you took the medication. If you still have LF, you will be treated again with the standard treatment for LF in Haiti (2 drugs). So in total, you will be in this study for one year. After we have finished distributing the drugs for LF, will ask some of you about how you felt about the study.

What are the risks of the study?

The risk of drawing blood from a finger prick is minimal, although some people become dizzy. You may experience momentary discomfort and/or bruising. Children may be uncomfortable and cry when blood is drawn. You will be watched by members of the research team and be given the opportunity to rest if you feel dizzy. Excess bleeding or infection may occur after a finger prick. The study personnel are trained in sterile techniques, so this chance is very small.

When you take the drugs your body may react to the dying worms by developing a fever. You may also feel tired or have body aches for one or two days. This means that the drugs are killing worms. Other possible effects are an upset stomach such as nausea after ingesting the

tablet, dizziness and occasionally itchy skin. If you experience any of these side effects, you will be seen by a medical personnel and refer to a physician if needed.

Are there benefits to taking part of the study?

This study will help treat your infection for LF and intestinal worms. The investigators hope that the information learned from this study will benefit people in Haiti as well as in other areas of the world affected by LF. From this work it may be possible to reduce the number of MDA (mass drug administration) treatments needed to eliminate this problem in Haiti.

Is the participation in this study voluntary?

You are free to choose to be in this study or not. Taking part of the study is voluntary. If you decide not to be in this study, you and your family will not lose any benefits. You may still be treated at the Community Health Center. If you agree to participate in this study, you may stop your participation at any time without any penalty. If you leave the study and the blood test shows that you are infected with LF, you will be referred to the local health center and be treated according to the standard treatment for LF in Haiti (2 drugs, DEC and ALB).

Are you going to share information you collected about me with other persons?

Only the people involved in this study will have access to the information collected in this study. Any information will be kept private as allowed by law. Your name will not appear in any report that comes from this survey. Your blood sample will not have any identifying information about you on it. We will share the test results obtained as part of the study with the local health worker at the health center only if you approve.

What if I become sick as a result of the study?

If you experience physical injury or illness as a result of participating in this study, medical care will be available at the local health center or the hospital. You will be transported to the Departmental Hospital if your symptoms are more severe. If your illness is determined to be related to taking the drugs for LF, we will cover all the medical costs.

What are you going to do with the samples of blood you collect?

A little more blood could be collected to be saved and stored in a freezer and send to the United States. Your blood may be used for future testing for LF or for other diseases that might affect the health of your community. Your blood will NOT be tested for HIV. We will not perform human genetic testing on your blood. Stored samples will be labelled with a code only and not linked to your name. Stored samples will only be used with approval from an Ethical Committee. You will not be contacted for additional consent. You may still participate in this study if you do not

consent to give additional blood for future scientific studies about diseases affecting your community.

How much does it cost to participate to the study?

There is no cost to you to participate in this study. You will receive no payment for taking part in this study. All study drugs and laboratory tests are free.

You are free to contact the following person for any details and clarifications:

Dr. Jean Frantz Lemoine, MD, MPH,
Coordonnateur de Programmes Nationaus de la Malaria et de la Filariose Lymphatique
Tel: (509) 3744-8755 or (509) 3946-3026

Project Title: Community Based Safety Study of 2-drug (Diethylcarbamazine and Albendazole) versus 3-drug (Ivermectin, Diethylcarbamazine and Albendazole) Therapy for Lymphatic Filariasis in Haiti

Part B : Information Sheet (children 7–17 year old, if a parent accepted that the child participates to the study)

We want to ask you to participate in a research study. We are currently investigating a new combination of three drugs against a disease called lymphatic filariasis (LF). LF is a disease carried by mosquitoes and caused by tiny worms in the blood. In Haiti we sometimes call it maladi filarioz. It can cause complications like swelling of the legs (lymphedema) or the scrotum (hydrocele). This new combination has been shown in other studies to be more effective in treating LF than the usual combination of two drugs (diethylcarbamazine and albendazole). The new combination consists of the usual drugs you take for LF (diethylcarbamazine and albendazole) plus another new drug to Haiti called ivermectin. This study is done to learn whether people feel worse if they have taken the three drugs together to get rid of the LF parasite.

If you accept to participate, we will ask you few questions about your health. It is possible that a nurse examine you briefly. We will also take some drops of blood from your finger tip to test for LF. Finally, we will ask you to swallow some tablets that treat LF. In total, it will take about 30 minutes of your time.

We will visit you several times in the 7 days after you have taken the tablets. During the first two days after you have taken the tables we will ask you how you feel. If you feel sick, we will ask you few more questions and we will examine you. During the next 5 following days, we will make sure that you still feel ok. If you feel sick, you will be examined by a nurse or a doctor.

When we prick your finger, it is possible that you will be afraid or that it will be a little painful. When you take the drugs your body may react to the dying worms by developing a fever. You may also feel tired or have body aches for one or two days. This means that the drugs are killing worms. Other possible effects are an upset stomach such as nausea after ingesting the tablet, dizziness and occasionally itchy skin. If you experience any of these side effects, you will be seen by a medical personnel and refer to a physician if needed.

You can refuse to participate to the study. It is not a problem at all. You can also change your mind during the study and stop to participate.

If you have questions, you can ask your Mom and Dad. You can also ask questions to any of the research team members. Do you have any questions?

You Dad and Mom accepted that you participate to the study. Do you accept to participate to the study?

Project Title: Community Based Safety Study of 2-drug (Diethylcarbamazine and Albendazole) versus 3-drug (Ivermectin, Diethylcarbamazine and Albendazole) Therapy for Lymphatic Filariasis in Haiti

Part C (Consent for adults)

Written Informed Consent Form for Participation in the Study (Adult ≥18 year old)

I have read or somebody has read to me the information included in the information sheet, and I fully understood the details. I have also been explained by the Principal Investigator or his duly authorized representative the full details. Having fully satisfied myself with the explanation given, of my own decision with full sense / awareness, give my consent for all screening procedures that are required for screening for infection due to LF. Also I give my consent for being treated with the assigned (2- or 3-drug combination) drugs for clearing infection.

I am also fully aware of my right to withdraw myself from the study, and in case should I stop participating, that I will be given treatment for the disease or will be referred for appropriate treatment.

Given this day____________in the month of _________________in the year_________
Signed in my presence.

Name of the individual:_____________________________

Signature of the individual:__________________________

Name of the witness:________________________

Signature of the witness: ___________________________

Finger print of individual if informed consent signed by witness

Address 1: ____
____

Address 2: ____
____

Name of the informant: ____

Signature of the informant: ____

Project Title: Community Based Safety Study of 2-drug (Diethylcarbamazine and Albendazole) versus 3-drug (Ivermectin, Diethylcarbamazine and Albendazole) Therapy for Lymphatic Filariasis in Haiti.

Part D (Consent for children)

Written Consent Form for Children (less than 18 year old) Participating in the Study

I have read or somebody has read to me the information included in the information sheet, and I fully understood the details. I have also been explained by the Principal Investigator or his duly authorized representative the full details, and having fully satisfied myself with the explanation given, of my own decision with full sense / awareness, give my consent for / my son / my daughter for all screening procedures that are required for screening for infection due to filariasis. Also I give my consent that my child will be treated with the assigned (2- or 3-drug combination) drugs for clearing infection. I am also fully aware of my right to withdraw my ward from the study, and in case should I stop my child participating, that my ward will be given treatment for the disease or will be referred for appropriate treatment.

Given this day____________in the month of _________________in the year_________
Signed in my presence.

Name of the parent/guardian: _______________________

Signature of the parent/guardian: ____________________

Name of the child: _____________________________

Name of the witness: ____________________________

Signature of the witness: __________________________

Finger print of parent/guardian if informed consent signed by witness

Address 1: ____
____

Address 2: ____
____

Name of the informant: ____

Signature of the informant: ____

Project Title: Community Based Safety Study of 2-drug (Diethylcarbamazine and Albendazole) versus 3-drug (Ivermectin, Diethylcarbamazine and Albendazole) Therapy for Lymphatic Filariasis in Haiti.

Part E (Assent)

<u>Assent for children 7–17 years old (only if a parent has accepted that the child participates)</u>

I have read or somebody has read to me the information included in the information sheet, and I fully understood the details. The Principal Investigator or his/her representative also explained to me the details of the study. I am satisfied with the explanation that was given to me. I agree to participate to the study.

Given this day____________in the month of _________________in the year_________
Signed in my presence.

Name of the individual: ___________________________________

Signature of the individual: ________________________________

Name of the witness: ______________________________

Signature of the witness: _________________________________

Finger print of individual if informed consent signed by witness

Address 1: ____
____

Address 2: ____
____

Name of the informant: ____

Signature of the informant: ____

Project title: Community Based Safety Study of 2-drug (Diethylcarbamazine and Albendazole) versus 3-drug (Ivermectin, Diethylcarbamazine and Albendazole) Therapy for Lymphatic Filariasis in Haiti.

Part F (Blood storage)

Consent for Future use of Participant's Blood

I have read or somebody has read to me the information included in the information sheet, and fully understood the details. I have also been explained by the Principal Investigator or his duly authorized representative the full details, and having fully satisfied myself with the explanation given, of my own decision with full sense / awareness, give my consent for my blood or the blood of my son / my daughter for storage and additional testing in the future.

Given this day____________in the month of _________________in the year_________
Signed in my presence.

Name of the individual or parent/guardian: ___________________

Signature of the individual or parent/guardian: ________________

Name of the child if less than 18 year old:____________________

Name of the witness: ________________________

Signature of the witness: ___________________________

Finger print of individual if informed consent signed by witness

Address 1: ____
____

Address 2: ____
____

Name of the informant: ____

Signature of the informant: ____
