# Supplementary material for: The safety of double- and triple-drug community mass drug administration for lymphatic filariasis: A multicenter, open-label, cluster-randomized study
Source: PLoS Med. 2019 Jun 24;16(6):e1002839. doi: 10.1371/journal.pmed.1002839 (PMC6590784; doi:10.1371/journal.pmed.1002839)
Supplement: S2 Appendix — (PDF) [file pmed.1002839.s003.pdf]

Data Analysis Plan

DOLF Triple Drug Therapy Studies

7/14/2016

Data Analysis Plan

Statistical analysis of data: Data analysis will focus separately on two dichotomous primary outcomes: the occurrence of serious adverse events (SAEs) and of AEs that are not serious. Unless otherwise stated below, analyses of AEs that are not serious will exclude grade one AEs because these mild events are likely to be primarily a source of noise in the data. Aside from the treatment group assignment, the key predictors of adverse events will be two dichotomous measures of infection, the results of the filariasis test strip (FST) and the presence of microfilariae (Mf). We will also be interested in the prognostic role of the Mf count in subjects with Mf infections at baseline. For the sake of simplicity in the discussion below, we will refer commonly to analyses focused on the prognostic significance of the presence of infection in settings where we will in practice perform two sets of analyses, one for FST infections and one for Mf infections.

One further point regarding the framework within which data analyses will be performed relates to the fact that this is a cluster randomized trial with the village as the unit of randomization. However, the large sample size and the expectation that an adverse event in a particular individual in a village will not influence the likelihood that a different individual in that village will experience an adverse event suggests that we will not require the use of the hierarchical models that are commonly employed in cluster trials. Nevertheless, as we note below, sensitivity analyses related to the primary hypotheses that do employ hierarchical models will be performed. These will facilitate an evaluation of our expectation that such models are not essential in this setting.

Primary analyses: With the above comments in mind, our primary analysis will involve chi square tests to compare SAE and non-serious AE rates in the two study arms. We will also compute confidence bounds on the between-group difference in SAE and non-serious AE rates. Logistic regression will be used to evaluate the impact of existing infection (FST and Mf) on SAE and AE rates and to explore the relationship between infection status and treatment group as predictors of adverse events. We will also look separately at results in subjects with and without infections of each of the two types and will be particularly interested in logistic models that evaluate the impact FST positivity has on adverse events in subjects who are Mf negative. In subjects who have Mf infections at baseline, we will use logistic models to evaluate the impact of the baseline Mf count on the SAE and non-serious AE rate. In analyses that include the Mf count, careful consideration will be given to the distributional properties of this variable, and since experience suggests that they will not be normally distributed, it is likely that the analyses will be performed following a data transformation to normality. In order to understand their impact on adverse events, we will include in the above analytic models additional covariates such as the age and sex of the subject. In all logistic regression analyses both in this section and below, the Hosmer-Lemeshow goodness of fit test will be used to evaluate the fit of the model.

Timing of preliminary analyses of AEs following treatment: Preliminary analyses will be conducted for each country study when data are available for half of the planned participant enrollment. Preliminary analysis will also be conducted with combined data from all

study sites when data are available for 10,000 persons treated with the triple drug (IDA) regimen. The preliminary analyses will be limited to chi square tests to compare SAE and non-serious AE rates in the two study arms and logistic regression analysis of relationships between infection status and treatment group as predictors of adverse events.

Sensitivity analyses: To confirm the conclusions generated by the above analyses, sensitivity analyses will be performed using generalized mixed models which account for the fact that the subject is nested within the village. This will be accomplished using PROC GLIMMIX in SAS with a logit link function and with village as a random effect. If results of these analyses are substantially different from the less complex procedures described above, we will use standard fit statistics to help us determine whether the mixed model results provide a better fit for the data.

Country effects: The above analyses will be performed separately using data from each country. They will also be performed with all countries included, a set of analyses that will include the country as a covariate. This will provide information about whether there are between-country differences in the efficacy of the three drug combination in comparison to standard treatment. Since the populations in some countries (India, Haiti, Sri Lanka) have received many prior rounds of mass treatment while other countries (PNG, Indonesia) have not received such treatment, we will also perform the analyses just described with a dichotomous covariate that measures whether mass treatment has been previously available. This will tell us whether the existence of prior mass treatment has an impact on the non-serious AE rate and the SAE rate.

Adverse events by grade: The outcome measures in the above analyses are either all SAEs or all non-serious AEs, independent of the grade of the event. To address questions of the role of the grade of the AE, we will first evaluate grade 1 adverse events to confirm our expectation that such AEs are primarily noise that will detract from our ability to evaluate the impact of the three drug intervention. This will be evaluated using chi square tests that determine whether there are differences in grade 1 AE rates in the two treatment arms. We will also place confidence bounds around the between-group difference in grade 1 AE rates. Other analyses will include grade 2, 3, and 4 AEs in the same analytic models. Since we expect very few if any deaths (a grade 5 AE), we will treat grade 4 and 5 AEs as a single category for the purposes of these analyses. To evaluate effects on AE grade, ordered logistic regression will be used with the grade of the AE as the 3-category outcome measure (grade 1 excluded). Predictor variables in these analytic models will include the treatment group, whether the subject was positive for infection at baseline, and the age and sex of the subject. These analyses will be performed (1) with data from each country evaluated separately and (2) using data from all countries in the same analysis, with the country being included as a covariate. This second set of analyses will also evaluate the interaction between treatment group and country as a predictor, with the interaction providing information about whether there are country by country differences in the effect of the three drug combination as compared to standard two drug therapy on AE rates.

Adverse events by study team: We expect that there will be approximately 1 field team for each thousand subjects enrolled in the study in most study sites. In order to evaluate whether there are differences between field teams in their identification of adverse events, the

following analyses will be conducted. Within each country, we will begin by performing simple chi square tests that evaluate whether SAE rates and non-serious AE rates differ by study team. These will be followed up with country-specific logistic regression models with SAEs and with non-serious AEs as the outcome. The predictor variables in these logistic models will include the study team along with the group assignment, the age and sex of the subject, and whether the subject had an infection at baseline. The latter analyses will tell us whether any differences in AE rates by study team can be explained, for example, by possible differences in the Mf rate. If we see study team differences in AE rates after covariate adjustment, it will suggest possible differences in protocol implementation from team to team and may provide insights into the training of these teams for future reference.

Efficacy of the two treatments: Efficacy data will be collected one year after subjects are treated to gather information on the clearance or persistence of filarial antigenemia by FST and on clearance/reduction of microfilaremia relative to baseline levels. Subjects included in these analyses will be those who were infected at baseline. Among those subjects, chi-square tests will tell us whether the rate of the clearance of infection differs by study group while logistic regression will determine whether there are between-group differences in the clearance rate after adjusting for age and sex. Among subjects with Mf infections at baseline, the impact on the Mf count of the three drug combination as compared to the two drug combination will be evaluated using analyses of covariance. In these analyses, the 1-year Mf count will be the dependent variable and the predictor variables will include the baseline Mf count, the study group, and the age and sex of the subject. In these linear models, the likely skewed distribution of the Mf count will probably require that data transformations be performed to improve the regression fit, with the appropriateness of any transformation being confirmed by evaluating regression residuals. All of these analyses will be conducted both within country and including all countries, with the country treated as a covariate in the model.
