# Supplementary material for: The safety of double- and triple-drug community mass drug administration for lymphatic filariasis: A multicenter, open-label, cluster-randomized study
Source: PLoS Med. 2019 Jun 24;16(6):e1002839. doi: 10.1371/journal.pmed.1002839 (PMC6590784; doi:10.1371/journal.pmed.1002839)
Supplement: S3 Appendix — (DOCX) [file pmed.1002839.s004.docx]

**S3 Appendix. Brief clinical summaries of serious adverse events reported after treatment**

| **SAE number 1**  **SAE Term**: | High Blood Pressure, Urinary tract infection and anemia | | | |
| --- | --- | --- | --- | --- |
| **CTCAE Grade:** | 3 | | | |
| **Serious Criteria**: | Inpatient or Prolonged Hospitalization | | | |
| **Treatment Arm**: | DEC+ALB | | **Treatment Date**: | Nov 28, 2016 |
| **Relationship of SAE to Treatment**: | | Possibly Related | | |
| **SAE Start Date**: | Dec 01, 2016 | | **SAE End Date**: | Dec 02, 2016 |
| **SAE Outcome**: | Recovered/Resolved | | | |
| **Sex**: | Male | | **Age**: | 78 |
| **Description:** | On December 1, 2016 a participant enrolled in the two-drug arm of the study in Haiti was hospitalized for fever, altered mental status (severe), high blood pressure, and a urinary tract infection 3 days after taking the study medication. The participant was admitted overnight and treated for a suspected urinary tract infection, high blood pressure, and anemia. During his hospitalization, the participant received Dipyrone (1000 mg), and Diazepam (one 25mg dose for agitation). The participant was discharged the day after admission. Discharge medications included enalapril (10mg daily), ciprofloxacin (500 mg twice per day for 10 days), ferrous sulfate, and vitamin C. The participant was well oriented when he left the hospital.  A urine culture was not performed because of incontinence. The study’s medical monitors suggested that the event be labeled “possibly related” to the study drug.  During follow-up at home on Dec 07, 2016 the participant was well oriented with normal vital signs. | | | |
| **SAE number 2**  **SAE Term**: | Dysuria, Nausea/vomiting, Fever | | | |
| **CTCAE Grade:** | 3 | | | |
| **Serious Criteria**: | Inpatient or Prolonged Hospitalization | | | |
| **Treatment Arm**: | DEC+ALB | | **Treatment Date**: | Nov 29, 2016 |
| **Relationship of SAE to Treatment**: | | Possibly Related | | |
| **SAE Start Date**: | Dec 05, 2016 | | **SAE End Date**: | Dec 06, 2016 |
| **SAE Outcome**: | Recovered/Resolved | | | |
| **Sex**: | Male | | **Age**: | 35 |
| **Description:** | On December 5, 2016 a participant enrolled in the two-drug arm of the study in Haiti was hospitalized for dysuria, nausea/vomiting, and fever 6 days after taking the study medication. The participant also complained of mild pain and swelling of the right testicle. The participant had a positive FTS result). The attending physician’s assessment was “possibly related” to study drugs. Nausea/vomiting may be secondary to gastritis; Genitourinary infection – r/o orchitis, epididymitis, STD.  The participant was treated with paracetamol 500 mg PO x 1 for fever, ORS rehydration. He was hospitalized overnight and treated with intravenous fluids, ranitidine 50 mg IV, dipyrone 1000 mg IM x 1, and paracetamol 500 mg PO x 1. The participant had 2 episodes of vomiting in 10 hours but improved the next morning, was afebrile, tolerating POs and feeling better. He was discharged on Dec 6 with prescriptions of amoxicillin 500 mg 2 tabs PO BID x 10 d; clarithromycin 500 mg PO BID x 10 d; omeprazole 20 mg PO BID; Vitamin B Complex 2 times PO BID; gastrogel susp 1 cc after each meal. UA/microscopy showed WBC 35-45/clumps; Bacteria ++++, Gram positive diplococci. | | | |

| **SAE number 3**  **SAE Term**: | Abdominal pain, soil-transmitted helminthiasis, *H. pylori* infection | | | |
| --- | --- | --- | --- | --- |
| **CTCAE Grade:** | 3 | | | |
| **Serious Criteria**: | Inpatient or Prolonged Hospitalization | | | |
| **Treatment Arm**: | DEC+ALB | | **Treatment Date**: | Dec 09, 2016 |
| **Relationship of SAE to Treatment**: | | Probably related | | |
| **SAE Start Date**: | Dec 10, 2016 | | **SAE End Date**: | Dec 11, 2016 |
| **SAE Outcome**: | Recovered/Resolved | | | |
| **Sex**: | Male | | **Age**: | 13 years |
| **Description:** | On December 10, 2016, one day after taking the study medication, a participant enrolled in the two-drug arm of the study in Haiti was hospitalized for severe abdominal pain, ascariasis, and *Helicobacter*  *pylori* infection. The participant’s mother stated that her son had passed a worm in his stools. Participant was evaluated at his home and was found to be afebrile, but complaining of mid-epigastric and periumbilical pain (grade 3) and unable to take POs. Participant was sent to hospital for further evaluation. At the hospital participant had T: 36.4 C, BP: 90/50, RR: 22, HR: 68, and complained of abdominal pain, nausea, vomiting, weakness and myalgia. Systems review was otherwise negative. Physical exam had tenderness to palpation in the periumbilical and epigastric areas.  Lab results included a positive test for antibodies to *Helicobacter pylori.* Hospital treatment included intravenous fluids, scopolamine 10 mg PO BID; and diclofenac 10 mg IV or IM q 8h. Participant spent ~20h in the hospital and improved with IV fluids and pain control. He had no episodes of nausea or vomiting in hospital, nor did he pass any more worms. His abdominal pain subsided, he was able to take POs and was discharged on Dec 11 with the following discharge diagnosis: SAE probably secondary to treatment that led to hospitalization, now resolved; STH infection without evidence of obstruction, presumed ascariasis; *H. pylori* positive. The participant was discharged with the following medications: amoxicillin 500 mg PO BID x 14 days; metronidazole suspension 250 mg/5 cc PO TID x 14 days; omeprazole 20 mg PO daily x 14 days; scopolamine 10 mg PO BID | | | |

| **SAE number 4**  **SAE Term**: | Dizziness | | | |
| --- | --- | --- | --- | --- |
| **CTCAE Grade:** | 2 | | | |
| **Serious Criteria**: | Inpatient or Prolonged Hospitalization | | | |
| **Treatment Arm**: | DEC+ALB | | **Treatment Date**: | Jul 21, 2017 |
| **Relationship of SAE to Treatment**: | | Possibly Related | | |
| **SAE Start Date**: | Jul 24, 2017 | | **SAE End Date**: | Jul 28, 2017 |
| **SAE Outcome**: | Recovered/Resolved | | | |
| **Sex**: | Female | | **Age**: | 68 |
| **Description:** | On July 27, 2017, six days after taking the study medication, the participant enrolled in the two-drug arm of the study in Fiji was hospitalized for severe dizziness. The participant had a previous diagnosis of hypertension, and her hypertension medications were last taken on July 18, 2017. On July 24, 2017 the participant was seen by a physician and complained of dizziness. Her blood pressure was normal during the July 24 visit. She reported that she had been feeling dizzy since before she took her MDA meds on July 21, 2017. She was seen again on the July 27, 2017 and complained of severe dizziness and was admitted with 1L IV fluids to run overnight and hypertension medicine was restarted. Hospitalization required that this event be reported as an SAE. She recovered fully and was discharged the next morning on Jul 28, 2017 with no complaints. | | | |

| **SAE number 5**  **SAE Term**: | Cellulitis | | | |
| --- | --- | --- | --- | --- |
| **CTCAE Grade:** | 3 | | | |
| **Serious Criteria**: | Persistence of significant disability/incapacity | | | |
| **Treatment Arm**: | IVM+DEC+ALB | | **Treatment Date**: | Aug 05, 2017 |
| **Relationship of SAE to Treatment**: | | Possibly Related | | |
| **SAE Start Date**: | Aug 10, 2017 | | **SAE End Date**: | Aug 31, 2017 |
| **SAE Outcome**: | Recovered/Resolved with sequelae | | | |
| **Sex**: | Female | | **Age**: | 74 |
| **Description:** | On August 12, 2017, seven days after taking the study medication, a participant enrolled in the three-drug arm of the study in Fiji reported severe fatigue, severe muscle pain, moderate nausea, and severe unusual swelling of the left leg. She had been unable to walk on the leg since August 10, 2017. The participant had a history of diabetes, hypertension, and for approximately 30 years, lymphedema of the left leg. She was prescribed oral flucloxacillin, ibuprofen and paracetamol. She was also treated with several daily doses of intramuscular procaine penicillin. Participant was managed as an outpatient based on limited systemic symptoms, notably no fever. There was no need for fluid support despite nausea and the participant tolerated oral fluids. Pain was managed with analgesics. The event was reported as a SAE because it was associated with prolonged disability (inability to walk for or perform activities of daily living). The participant took her last dose of flucloxacillin August 31, 2017. Her lymphedema returned to baseline by September 5, 2017, but her mobility was limited by her deconditioned state. She was last followed up on August 7, 2018. At that time, she reported that her diability had completely resolved on November 1, 2017. | | | |

| **SAE number 6**  **SAE Term**: | Acute exacerbation of COPD | | | |
| --- | --- | --- | --- | --- |
| **CTCAE Grade:** | 3 | | | |
| **Serious Criteria**: | Inpatient or Prolonged Hospitalization | | | |
| **Treatment Arm**: | DEC+ALB | | **Treatment Date**: | Aug 21, 2017 |
| **Relationship of SAE to Treatment**: | | Possibly Related | | |
| **SAE Start Date**: | Aug 21, 2017 | | **SAE End Date**: | Aug 26, 2017 |
| **SAE Outcome**: | Recovering/Resolving | | | |
| **Sex**: | Male | | **Age**: | 52 |
| **Description:** | On August 22, 2017, one day after taking the study a participant enrolled in the two-drug arm of the study in Fiji complained of dizziness and joint pains but was able to mobilize without assistance and his symptoms did not interfere with daily activities. The participant received 2 salbutamol nebulizer treatments and 3 saline nebulizer treatments and was sent home after his symptoms settled. On August 23, 2017 he was still having difficulty sleeping and was admitted to the hospital for closer monitoring and 2 hourly nebulizers. This case was reported as an SAE because of the hospital admission. He was discharged on August 26 with salbutamol inhalers as needed and paracetamol for joint pain.  The participant had a known history of COPD and had been admitted to hospital for exacerbation of COPD several times since 2015. His most recent hospitalization was on July 20-27, 2017.  The relationship of the study drug to the SAE was determined as possibly related (impossible to rule out). Respiratory symptoms can occur after treatment of lymphatic filariasis with DEC. The participant had a positive filarial antigen test and had 262 microfilariae per 60 µl of blood prior to treatment. However, his long history of COPD and hospitalization one month prior to treatment made it difficult to know whether anti-filarial treatment caused this SAE. | | | |
