# Supplementary material for: The safety of double- and triple-drug community mass drug administration for lymphatic filariasis: A multicenter, open-label, cluster-randomized study
Source: PLoS Med. 2019 Jun 24;16(6):e1002839. doi: 10.1371/journal.pmed.1002839 (PMC6590784; doi:10.1371/journal.pmed.1002839)
Supplement: S1 Table — (DOCX) [file pmed.1002839.s005.docx]

**S1 Table. Guide for Assigning Adverse Event Severity**

Guide for Assigning Adverse Event Severity Grades 1-4

G*rade 0 = no symptoms; grade 5 = death from adverse event*

**Note on general aspects of grading**

**0** = No adverse event or within normal limits

**1** = Mild adverse event, does not interfere with work or school

**2** = Moderate adverse event, interferes with work or school at least 1 day

**3** = Severe and undesirable adverse event; interferes with ADL, requires medical assessment

**4** = Potentially life-threatening or disabling adverse event; requires transfer to medical facility

**5** = Death

Any event grade of 2 requires vital signs on the participant.

Any event > grade 3 requires a medical evaluation and notification of the medical officer. Any grade 3, 4 or 5 event or overnight hospitalization requires a Serious Adverse Event Report.

| **Symptoms/Signs** | **Grades** | | | |
| --- | --- | --- | --- | --- |
|  | **1. Mild** | **2. Moderate** | **3. Severe** | **4. Life-threatening** |
| Fever (non-axillary temperatures only) | 38.0 – 39.0ºC | 39.1 – 40.0ºC | > 40.0ºC | > 40.0ºC for > 48 hrs |
| Headache | Mild pain not interfering with work or school | Moderate pain; pain or analgesics interfering with ability to work or attend school | Severe pain; pain or analgesics interfering with activities of daily living | Disabling, duration > 48 hrs. |
| Nausea | Able to eat | Oral intake significantly decreased | No significant intake, requiring IV fluids | - |
| Vomiting | 1 episode in 24 hours over pretreatment | 2-5 episodes in 24 hours over pretreatment | ≥ 6 episodes in 24hours, or need for IV fluids (0utpatient) | Hemodynamic collapse or overnight hospitalization |
| Diarrhea | Increase of < 4 stools/day over pre-treatment | Increase of 4-6 stools/ day, or nocturnal stools | Increase of ≥ 7 stools/ day or need for outpatient parenteral support for dehydration | Physiologic consequences with hemodynamic collapse or requiring hospitalization |
| Abdominal pain | Mild pain not interfering with work or school | Moderate pain; pain or analgesics interfering with ability to work or attend school | Severe pain; pain or analgesics interfering with activities of daily living | Disabling, duration > 48 hrs. |
| Acute swelling (beyond baseline lymphedema) | Mild, not interfering with work or school | Moderate, unable to work or attend school 1 day | Severe, unable to work/school >1 day | Severe, limiting activities of daily living (unable to walk) > 2 days |
| Joint or muscle pain | Mild pain not interfering with work or school | Moderate pain; pain or analgesics interfering with ability to work or attend school | Severe pain; pain or analgesics interfering with activities of daily living | Disabling, duration > 48 hrs. |
| Swollen or painful nodes (armpit or groin)* | Mild, not interfering with work or school | Moderate, unable to work or attend school 1 day | Severe, unable to work or attendschool >1 day | Severe, limiting activities of daily living (unable to walk) > 2 days |
| Men only: testicular or scrotal pain | Mild, not interfering with work or school | Moderate, unable to work or attend school 1 day | Severe, unable to work or attend school >1 day | Severe, limiting activities of daily living (unable to walk) > 2 days |
| Rash | Localized rash (covers only one part of the body) | Diffuse rash (covers multiple parts of the body) | Diffuse rash (covers multiple parts of the body) AND has any blisters or ulcers or mouth sores | Extensive areas with blisters or ulcers OR peeling or blackening of skin |
| Itching skin | Mild, not interfering with work or school | Moderate, unable to work or attend school 1 day | Severe, unable to work/school >1 day |  |
| Cough | Mild, relieved by non-prescription medication | Requiring narcotic antitussive | Severe cough or coughing spasms, poorly controlled by treatment | Hospitalization or respiratory failure requiring mechanical ventilation |
| Difficulty breathing (wheezing or dyspnea) | Mild, not interfering with work or school | Moderate, unable to work or attend school for 1 day | Severe, more than 1 day and required transfer to clinic or hospital | Hospitalization or respiratory failure requiring mechanical ventilation |
| Fatigue | Mild, not interfering with work or school | Moderate, unable to work or attend school at least 1 day | Unable to perform activities of daily living, > 1day | Required hospitalization |
| Dizziness, giddiness, or fainting | Mild, not interfering with work or school | Moderate, unable to work or attend school for 1 day, but no fainting | Any loss of consciousness (fainting) | - |
| Confusion or excess drowsiness* | Mild, not interfering with work or school | Moderate; confusion or drowsiness interfering with ability to work | Confusion, loss of memory, or sleepiness interfering with activities of daily living | Delirium, inability to rouse, or coma |
| Other illness or symptoms | Mild, not interfering with work or school | Moderate, unable to work or attend school at least 1 day | Unable to perform activities of daily living, > 1day | Required hospitalization |
