# Supplementary material for: The safety of double- and triple-drug community mass drug administration for lymphatic filariasis: A multicenter, open-label, cluster-randomized study
Source: PLoS Med. 2019 Jun 24;16(6):e1002839. doi: 10.1371/journal.pmed.1002839 (PMC6590784; doi:10.1371/journal.pmed.1002839)
Supplement: S2 Table — (DOCX) [file pmed.1002839.s006.docx]

**S2. Table. Rates of follow-up for assessment of adverse events after**

**treatment^a^**

| **Variable** | **Drug regimen** | **Found: n/Total (%)** |
| --- | --- | --- |
| Participant found on visit 1 | DA | 11403/12280 (92.9) |
|  | IDA | 13908/14551 (95.6) |
| Participant found on visit 2 | DA | 10955/12280 (89.2) |
|  | IDA | 13678/14551 (94.0) |
| Participant found on visit 1 AND 2 | DA | 10504/12280 (85.5) |
|  | IDA | 13266/14551 (91.2) |
| Participant found on visit 1 OR 2 | DA | 11854/12280 (96.5) |
|  | IDA | 14320/14551 (98.4) |

^a^ Five subjects with missing data for day 1 or day 2 are not included

in the table calculations. Follow-up rates were significantly different between treatment groups for all variables (*P* < 0.001).
