# Supplementary material for: The safety of double- and triple-drug community mass drug administration for lymphatic filariasis: A multicenter, open-label, cluster-randomized study
Source: PLoS Med. 2019 Jun 24;16(6):e1002839. doi: 10.1371/journal.pmed.1002839 (PMC6590784; doi:10.1371/journal.pmed.1002839)
Supplement: S3 Table — (DOCX) [file pmed.1002839.s007.docx]

| **Symptom** | **Grade 1 AE** | **Grade 2 AE** | **Grade 3 AE** | **Grade 4 AE** | **All grades** |
| --- | --- | --- | --- | --- | --- |
| Headache | 404 (19) | 44 (25) | 2 (10) | 0 | 450 (20) |
| Dizziness | 252 (12) | 13 (7) | 1 (5) | 0 | 266 (12) |
| Fatigue | 199 (10) | 10 (6) | 1 (5) | 0 | 210 (9) |
| Fever | 158 (8) | 36 (20) | 2 (10) | 0 | 196 (9) |
| Abdominal pain | 165 (8) | 7 (4) | 4 (20) | 0 | 176 (8) |
| Nausea | 160 (8) | 5 (3) | 0 | 0 | 165 (7) |
| Joint pain | 90 (4) | 12 (7) | 0 | 0 | 102 (4) |
| Muscle pain | 87 (4) | 8 (4) | 1 (5) | 0 | 96 (4) |
| Muscle weakness | 91 (4) | 3 (2) | 0 | 0 | 94 (4) |
| Vomiting | 72 (3) | 8 (4) | 0 | 0 | 80 (3) |
| Itchy skin | 60 (3) | 6 (3) | 0 | 0 | 66 (3) |
| Diarrhea | 50 (2) | 5 (3) | 1 (5) | 0 | 56 (2) |
| Cough | 50 (2) | 3 (2) | 0 | 0 | 53 (2) |
| Scrotal pain or swelling | 32 (2) | 2 (1) | 1 (5) | 0 | 35 (2) |
| Rash | 22 (1) | 1 (1) | 1 (5) | 0 | 24 (1) |
| Difficulty breathing | 13 (1) | 1 (1) | 2 (10) | 0 | 16 (1) |
| Pelvic pain | 13 (1) | 0 | 0 | 0 | 13 (1) |
| Epigastric pain | 12 (1) | 0 | 0 | 0 | 12 (1) |
| Hypersalivation | 9 (0) | 0 | 0 | 0 | 9 (0) |
| Axillary pain | 7 (0) | 0 | 0 | 0 | 7 (0) |
| Groin swelling | 7 (0) | 0 | 0 | 0 | 7 (0) |
| Hypertension | 1 (0) | 2 (1) | 4 (20) | 0 | 7 (0) |
| Low back pain | 6 (0) | 0 | 0 | 0 | 6 (0) |
| Backache | 5 (0) | 0 | 0 | 0 | 5 (0) |
| Groin pain | 5 (0) | 0 | 0 | 0 | 5 (0) |
| Hunger | 5 (0) | 0 | 0 | 0 | 5 (0) |
| Leg pain | 5 (0) | 0 | 0 | 0 | 5 (0) |
| Sore throat | 5 (0) | 0 | 0 | 0 | 5 (0) |
| Swelling | 4 (0) | 1 (1) | 0 | 0 | 5 (0) |
| Drowsiness | 4 (0) | 0 | 0 | 0 | 4 (0) |
| Swelling of feet | 4 (0) | 0 | 0 | 0 | 4 (0) |
| Boil on leg | 3 (0) | 0 | 0 | 0 | 3 (0) |
| Eye swelling | 2 (0) | 1 (1) | 0 | 0 | 3 (0) |
| Insomnia | 2 (0) | 1 (1) | 0 | 0 | 3 (0) |
| Malaise | 3 (0) | 0 | 0 | 0 | 3 (0) |
| Neck pain | 2 (0) | 1 (1) | 0 | 0 | 3 (0) |
| Swelling (r) testicle | 3 (0) | 0 | 0 | 0 | 3 (0) |
| Swelling arm | 3 (0) | 0 | 0 | 0 | 3 (0) |
| Swelling of legs | 2 (0) | 1 (1) | 0 | 0 | 3 (0) |
| Testicular pain | 3 (0) | 0 | 0 | 0 | 3 (0) |
| Bloating | 2 (0) | 0 | 0 | 0 | 2 (0) |
| Breast swelling | 2 (0) | 0 | 0 | 0 | 2 (0) |
| Chest pain | 2 (0) | 0 | 0 | 0 | 2 (0) |
| Chills | 2 (0) | 0 | 0 | 0 | 2 (0) |
| Febrile reaction | 2 (0) | 0 | 0 | 0 | 2 (0) |
| Generalized aching | 2 (0) | 0 | 0 | 0 | 2 (0) |
| Hypogastric pain | 2 (0) | 0 | 0 | 0 | 2 (0) |
| Lumbar pain | 2 (0) | 0 | 0 | 0 | 2 (0) |
| Neck swelling | 2 (0) | 0 | 0 | 0 | 2 (0) |
| Rhinorrhea | 1 (0) | 1 (1) | 0 | 0 | 2 (0) |
| Throat pain | 2 (0) | 0 | 0 | 0 | 2 (0) |
| Ankle injury | 1 (0) | 0 | 0 | 0 | 1 (0) |
| Arm discomfort | 1 (0) | 0 | 0 | 0 | 1 (0) |
| Axillary lump | 1 (0) | 0 | 0 | 0 | 1 (0) |
| Blood pressure | 0 | 1 (1) | 0 | 0 | 1 (0) |
| Boil | 1 (0) | 0 | 0 | 0 | 1 (0) |
| Boil on face | 1 (0) | 0 | 0 | 0 | 1 (0) |
| Breast abscess | 1 (0) | 0 | 0 | 0 | 1 (0) |
| Breast inflammation | 1 (0) | 0 | 0 | 0 | 1 (0) |
| Breast tenderness | 1 (0) | 0 | 0 | 0 | 1 (0) |
| Bruise | 1 (0) | 0 | 0 | 0 | 1 (0) |
| Cervical pain | 1 (0) | 0 | 0 | 0 | 1 (0) |
| Common cold | 1 (0) | 0 | 0 | 0 | 1 (0) |
| Conjunctivitis | 0 | 1 (1) | 0 | 0 | 1 (0) |
| Constipation | 1 (0) | 0 | 0 | 0 | 1 (0) |
| Dysmenorrhea | 1 (0) | 0 | 0 | 0 | 1 (0) |
| Dysuria | 1 (0) | 0 | 0 | 0 | 1 (0) |
| Ear ache | 1 (0) | 0 | 0 | 0 | 1 (0) |
| Ear pain | 1 (0) | 0 | 0 | 0 | 1 (0) |
| Epigastralgia | 1 (0) | 0 | 0 | 0 | 1 (0) |
| External ear inflammation | 1 (0) | 0 | 0 | 0 | 1 (0) |
| Eye redness | 0 | 1 (1) | 0 | 0 | 1 (0) |
| Eyebrow swelling | 1 (0) | 0 | 0 | 0 | 1 (0) |
| Facial pain | 1 (0) | 0 | 0 | 0 | 1 (0) |
| Facial swelling | 0 | 1 (1) | 0 | 0 | 1 (0) |
| Flu | 1 (0) | 0 | 0 | 0 | 1 (0) |
| General malaise | 1 (0) | 0 | 0 | 0 | 1 (0) |
| General pruritus | 1 (0) | 0 | 0 | 0 | 1 (0) |
| Gingival pain | 1 (0) | 0 | 0 | 0 | 1 (0) |
| Gum bleeding | 0 | 1 (1) | 0 | 0 | 1 (0) |
| Haemoptysis | 1 (0) | 0 | 0 | 0 | 1 (0) |
| Hearing reduced | 1 (0) | 0 | 0 | 0 | 1 (0) |
| Hypermenorrhea | 1 (0) | 0 | 0 | 0 | 1 (0) |
| Inappetence | 1 (0) | 0 | 0 | 0 | 1 (0) |
| Knee injury | 0 | 1 (1) | 0 | 0 | 1 (0) |
| Knee pain | 1 (0) | 0 | 0 | 0 | 1 (0) |
| Laceration of leg | 1 (0) | 0 | 0 | 0 | 1 (0) |
| Localized itching | 1 (0) | 0 | 0 | 0 | 1 (0) |
| Lymphadenopathy inguinal | 0 | 1 (1) | 0 | 0 | 1 (0) |
| Numbness | 1 (0) | 0 | 0 | 0 | 1 (0) |
| Numbness generalised | 1 (0) | 0 | 0 | 0 | 1 (0) |
| Numbness in leg | 1 (0) | 0 | 0 | 0 | 1 (0) |
| Numbness of upper arm | 1 (0) | 0 | 0 | 0 | 1 (0) |
| Pain in limb | 1 (0) | 0 | 0 | 0 | 1 (0) |
| Painful urination | 1 (0) | 0 | 0 | 0 | 1 (0) |
| Rigors | 1 (0) | 0 | 0 | 0 | 1 (0) |
| Sleepy | 1 (0) | 0 | 0 | 0 | 1 (0) |
| Swelling (l) testicle | 1 (0) | 0 | 0 | 0 | 1 (0) |
| Swelling face | 1 (0) | 0 | 0 | 0 | 1 (0) |
| Tingling tongue | 1 (0) | 0 | 0 | 0 | 1 (0) |
| Tonsillitis | 1 (0) | 0 | 0 | 0 | 1 (0) |
| Tooth pain | 1 (0) | 0 | 0 | 0 | 1 (0) |
| Weakness | 1 (0) | 0 | 0 | 0 | 1 (0) |
| Weakness generalized | 1 (0) | 0 | 0 | 0 | 1 (0) |
| Weakness of limbs | 1 (0) | 0 | 0 | 0 | 1 (0) |

| **Total grade 1** | **Total grade 2** | **Total grade 3** | **Total grade 4** | **Total all grades** |
| --- | --- | --- | --- | --- |
| 2088 | 179 | 20 | 0 | 2287 |

| **Symptom** | **Grade 1 AE** | **Grade 2 AE** | **Grade 3 AE** | **Grade 4 AE** | **All grades** |
| --- | --- | --- | --- | --- | --- |
| Headache | 503 (19) | 57 (23) | 1 (6) | 0 | 561 (20) |
| Dizziness | 303 (12) | 16 (7) | 2 (12) | 0 | 321 (11) |
| Fatigue | 302 (12) | 15 (6) | 2 (12) | 0 | 319 (11) |
| Fever | 257 (10) | 34 (14) | 0 | 0 | 291 (10) |
| Muscle weakness | 178 (7) | 7 (3) | 0 | 0 | 185 (6) |
| Abdominal pain | 164 (6) | 14 (6) | 2 (12) | 0 | 180 (6) |
| Muscle pain | 145 (6) | 13 (5) | 2 (12) | 0 | 160 (6) |
| Joint pain | 129 (5) | 16 (7) | 0 | 0 | 145 (5) |
| Nausea | 123 (5) | 12 (5) | 0 | 0 | 135 (5) |
| Vomiting | 76 (3) | 16 (7) | 1 (6) | 0 | 93 (3) |
| Diarrhea | 75 (3) | 7 (3) | 3 (18) | 0 | 85 (3) |
| Itchy skin | 75 (3) | 6 (2) | 0 | 0 | 81 (3) |
| Cough | 63 (2) | 5 (2) | 0 | 0 | 68 (2) |
| Rash | 24 (1) | 2 (1) | 0 | 0 | 26 (1) |
| Scrotal pain or swelling | 15 (1) | 1 (0) | 0 | 0 | 16 (1) |
| Difficulty breathing | 11 (0) | 2 (1) | 0 | 0 | 13 (0) |
| Groin swelling | 13 (0) | 0 | 0 | 0 | 13 (0) |
| Swelling of legs | 7 (0) | 3 (1) | 1 (6) | 0 | 11 (0) |
| Low back pain | 9 (0) | 0 | 0 | 0 | 9 (0) |
| Swelling arm | 9 (0) | 0 | 0 | 0 | 9 (0) |
| Drowsiness | 7 (0) | 0 | 0 | 0 | 7 (0) |
| Epigastric pain | 7 (0) | 0 | 0 | 0 | 7 (0) |
| Groin pain | 7 (0) | 0 | 0 | 0 | 7 (0) |
| Swelling | 6 (0) | 1 (0) | 0 | 0 | 7 (0) |
| Chills | 5 (0) | 0 | 1 (6) | 0 | 6 (0) |
| Sore throat | 5 (0) | 1 (0) | 0 | 0 | 6 (0) |
| Swelling of eyelid | 4 (0) | 2 (1) | 0 | 0 | 6 (0) |
| Sleepy | 5 (0) | 0 | 0 | 0 | 5 (0) |
| Hypersalivation | 4 (0) | 0 | 0 | 0 | 4 (0) |
| Common cold | 2 (0) | 1 (0) | 0 | 0 | 3 (0) |
| Hunger | 3 (0) | 0 | 0 | 0 | 3 (0) |
| Pain | 3 (0) | 0 | 0 | 0 | 3 (0) |
| Abscess leg | 2 (0) | 0 | 0 | 0 | 2 (0) |
| Axillary pain | 2 (0) | 0 | 0 | 0 | 2 (0) |
| Backache | 1 (0) | 1 (0) | 0 | 0 | 2 (0) |
| Chest pain | 1 (0) | 1 (0) | 0 | 0 | 2 (0) |
| Earache | 2 (0) | 0 | 0 | 0 | 2 (0) |
| Eye pain | 2 (0) | 0 | 0 | 0 | 2 (0) |
| Gastritis | 2 (0) | 0 | 0 | 0 | 2 (0) |
| Insomnia | 2 (0) | 0 | 0 | 0 | 2 (0) |
| Malaise | 2 (0) | 0 | 0 | 0 | 2 (0) |
| Numbness in hand | 2 (0) | 0 | 0 | 0 | 2 (0) |
| Pelvic pain | 1 (0) | 1 (0) | 0 | 0 | 2 (0) |
| Subcutaneous nodule | 1 (0) | 1 (0) | 0 | 0 | 2 (0) |
| Swelling abdomen | 2 (0) | 0 | 0 | 0 | 2 (0) |
| Visual impairment | 2 (0) | 0 | 0 | 0 | 2 (0) |
| Abscess | 1 (0) | 0 | 0 | 0 | 1 (0) |
| Ache nos | 1 (0) | 0 | 0 | 0 | 1 (0) |
| Ankle swelling | 1 (0) | 0 | 0 | 0 | 1 (0) |
| Appetite lost | 1 (0) | 0 | 0 | 0 | 1 (0) |
| Axillary lump | 1 (0) | 0 | 0 | 0 | 1 (0) |
| Blood pressure high | 1 (0) | 0 | 0 | 0 | 1 (0) |
| Boil on leg | 1 (0) | 0 | 0 | 0 | 1 (0) |
| Breast swelling | 0 | 1 (0) | 0 | 0 | 1 (0) |
| Conjunctivitis | 0 | 1 (0) | 0 | 0 | 1 (0) |
| Decreased appetite | 1 (0) | 0 | 0 | 0 | 1 (0) |
| Delusion | 0 | 1 (0) | 0 | 0 | 1 (0) |
| Dysentery | 0 | 0 | 1 (6) | 0 | 1 (0) |
| Dyspepsia | 1 (0) | 0 | 0 | 0 | 1 (0) |
| Ear pain | 1 (0) | 0 | 0 | 0 | 1 (0) |
| Ear ringing | 1 (0) | 0 | 0 | 0 | 1 (0) |
| Falciparum malaria | 0 | 1 (0) | 0 | 0 | 1 (0) |
| Febrile reaction | 1 (0) | 0 | 0 | 0 | 1 (0) |
| Fingers stiffness | 1 (0) | 0 | 0 | 0 | 1 (0) |
| Flu-like symptoms | 1 (0) | 0 | 0 | 0 | 1 (0) |
| Foot injury | 0 | 1 (0) | 0 | 0 | 1 (0) |
| Foot pain | 0 | 1 (0) | 0 | 0 | 1 (0) |
| Furuncle | 1 (0) | 0 | 0 | 0 | 1 (0) |
| Gastric pain | 1 (0) | 0 | 0 | 0 | 1 (0) |
| Generalized pruritus | 1 (0) | 0 | 0 | 0 | 1 (0) |
| Greenstick fracture | 0 | 1 (0) | 0 | 0 | 1 (0) |
| Head cold | 1 (0) | 0 | 0 | 0 | 1 (0) |
| Hemoptysis | 0 | 1 (0) | 0 | 0 | 1 (0) |
| Hemorrhoids | 1 (0) | 0 | 0 | 0 | 1 (0) |
| Hypogastric pain | 1 (0) | 0 | 0 | 0 | 1 (0) |
| Incision site infection | 1 (0) | 0 | 0 | 0 | 1 (0) |
| Infected skin ulcer | 1 (0) | 0 | 0 | 0 | 1 (0) |
| Influenza | 1 (0) | 0 | 0 | 0 | 1 (0) |
| Knee pain | 1 (0) | 0 | 0 | 0 | 1 (0) |
| Leg pain | 1 (0) | 0 | 0 | 0 | 1 (0) |
| Lip swelling | 0 | 1 (0) | 0 | 0 | 1 (0) |
| Lumbar pain | 1 (0) | 0 | 0 | 0 | 1 (0) |
| Malaria | 0 | 1 (0) | 0 | 0 | 1 (0) |
| Neck pain | 1 (0) | 0 | 0 | 0 | 1 (0) |
| Neck swelling | 1 (0) | 0 | 0 | 0 | 1 (0) |
| Pain in heel | 1 (0) | 0 | 0 | 0 | 1 (0) |
| Polyuria | 1 (0) | 0 | 0 | 0 | 1 (0) |
| Pruritis | 1 (0) | 0 | 0 | 0 | 1 (0) |
| Per vagina bleeding | 1 (0) | 0 | 0 | 0 | 1 (0) |
| Rigors | 0 | 0 | 1 (6) | 0 | 1 (0) |
| Scabies | 1 (0) | 0 | 0 | 0 | 1 (0) |
| Somnolence | 1 (0) | 0 | 0 | 0 | 1 (0) |
| Spontaneous penile erection | 1 (0) | 0 | 0 | 0 | 1 (0) |
| Sputum bloody | 1 (0) | 0 | 0 | 0 | 1 (0) |
| Stomach burning sensation of | 1 (0) | 0 | 0 | 0 | 1 (0) |
| Swelling (r) testicle | 1 (0) | 0 | 0 | 0 | 1 (0) |
| Swelling face | 1 (0) | 0 | 0 | 0 | 1 (0) |
| Swelling of feet | 1 (0) | 0 | 0 | 0 | 1 (0) |
| Swelling of hands | 1 (0) | 0 | 0 | 0 | 1 (0) |
| Swollen eyelid | 1 (0) | 0 | 0 | 0 | 1 (0) |
| Testicular swelling | 1 (0) | 0 | 0 | 0 | 1 (0) |
| Weakness | 1 (0) | 0 | 0 | 0 | 1 (0) |

| **Total grade 1** | **Total grade 2** | **Total grade 3** | **Total grade 4** | **Total all grades** |
| --- | --- | --- | --- | --- |
| 2607 | 245 | 17 | 0 | 2869 |

| \|  \| \| --- \| \| Note: These tables include all adverse events (AE’s) including all events counted as “other” in Figure 3. Some participants experienced more than one adverse event. For participants that had the same adverse event over multiple days, only the highest grade event is listed reported. AE’s are reported according to adverse event lowest level term (AELLT) medical coding. Serious adverse events are described in the S4 Appendix and not included in this table. Values reported are number and %, where % refers to the percentage of the total number of that type of AE by grade. \| \|  \| \|  \| |
| --- | --- | --- | --- | --- |
